# Effects of sixty six adolescent tobacco use cessation trials and seventeen prospective studies of self-initiated quitting

**DOI:** 10.1186/1617-9625-1-5

**Published:** 2002-01-15

**Authors:** S Sussman

**Affiliations:** 1 Institute for Health Promotion and Disease Prevention Research, University of Southern California, USA; 2 Professor of Preventive Medicine and Psychology and Institute for Health Promotion and Disease Prevention Research, University of Southern California/IPR, 1000 South Fremont, Unit 8, (Room 4124 in Building A-4), Alhambra, CA 91803, USA

## Abstract

This paper provides a review of the last two and a half decades of research in adolescent and young-adult tobacco use cessation. A total of 66 tobacco cessation intervention studies – targeted or population – are reviewed. In addition, an exhaustive review is completed of adolescent self-initiated tobacco use cessation, involving 17 prospective survey studies.

Average reach and retention across the intervention studies was 61% and 78%, respectively, and was higher when whole natural units were treated (e.g., classrooms), than when units created specifically for the program were treated (e.g., school-based clinics). The mean quit-rate at a three to 12-month average follow-up among the program conditions was 12%, compared to approximately 7% across control groups. A comparison of intervention theories revealed that motivation enhancement (19%) and contingency-based reinforcement (16%) programs showed higher quit-rates than the overall intervention cessation mean. Regarding modalities (channels) of change, classroom-based programs showed the highest quit rates (17%). Computer-based (expert system) programs also showed promise (13% quit-rate), as did school-based clinics (12%).

There was a fair amount of missing data and wide variation on how data points were measured in the programs' evaluations. Also, there were relatively few direct comparisons of program and control groups. Thus, it would be difficult to conduct a formal meta-analysis on the cessation programs. Still, these data suggest that use of adolescent tobacco use cessation interventions double quit rates on the average.

In the 17 self-initiated quitting survey studies, key predictors of quitting were living in a social milieu that is composed of fewer smokers, less pharmacological or psychological dependence on smoking, anti-tobacco beliefs (e.g., that society should step in to place controls on smoking) and feeling relatively hopeful about life. Key variables relevant to the quitting process may include structuring the context of programming for youth, motivating quit attempts and reducing ambivalence about quitting, and making programming enjoyable as possible. There also is a need to help youth to sustain a quit-attempt. In this regard, one could provide ongoing support during the acute withdrawal period and teach youth social/life skills. Since there is little information currently available on use of nicotine replacement in young people, continued research in this arena might also be a useful focus for future work.

## Introduction

Most adolescents who use tobacco regularly (e.g., monthly or greater) continue use into adulthood. For example, while only five percent of adolescent smokers view themselves as smoking five years later, 75% actually are smoking eight years later [1]. Risk for developing tobacco-related disease increases as a function of duration of time tobacco is used [2,3]. Thus, adolescent users are at particularly high risk for physical consequences of tobacco use later on. These consequences begin their course in adolescence (e.g., HDL level changes; [4]). Tobacco use prevalence among youth generally has been increasing over the last 15 years [3]. Until the last five years, little research has been completed to find ways to support young tobacco users to quit [5]. Possibly, people assumed that efforts aimed at getting teens to quit smoking simply don't work. However, the research and practice climates have changed. Ongoing efforts to provide teen cessation interventions have been initiated by numerous organizations. In the United States these organizations include the Centers for Disease Control, American Medical Association, National Institutes of Health (e.g., NCI, NIDA, NHLBI), American Lung Association, American Heart Association, and American Cancer Society [e.g., [6-8]]. In Canada, these include Health Canada as well as various other Canadian governmental and private organizations. Numerous other countries are involved in similar efforts (e.g., Australia, India, Finland, Korea, Nigeria, and the UK; [e.g., [9,10]]). Adolescent tobacco use cessation promises to arrest the physical consequences of use in a rapidly growing and developing body, and before the addiction becomes so ingrained that cessation becomes a much more difficult problem [11].

The present paper reviews adolescent tobacco cessation research completed to date. It builds on a previous review completed by Sussman, Lichtman, Ritt, & Pallonen [12]. This paper examines numerous types of teen cessation efforts. These efforts include not only clinic programs, but also classroom-based efforts, computer expert system interventions, family programs, policy efforts (e.g., price increases, smoke-free areas, access reduction programs), mass media programming, and State-wide programs (there were two such programs, one was a mass media campaign and the other was multi-component). All of these efforts are referred to herein as "programs, " in that they are organized programmatic efforts to produce a cessation or reduction effect. The term "intervention" is also used and is interchangeable with "programs." A total of 66 teen cessation programs are reviewed. Theories, modalities, methods, and results of these studies are provided. Examination of outcomes as a function of gender and ethnicity, theory-type, delivery modality, and number of sessions also is completed. A ranking of the evidence presented by the studies, based on outcomes and methodology, also is provided.

In addition, this paper provides a review of all known prospective self-initiated cessation survey studies. These are studies in which survey data on tobacco users is collected at two or more time-points. At baseline, tobacco users report various demographic, psychosocial, and behavioral sources of information. For example, they may report their gender and ethnicity, intention to quit tobacco use at a later time-point, and the number of cigarettes they smoke each day. These tobacco users are then surveyed at a later time-point. If these persons are found to have stopped smoking at the later time point, they are considered to have exhibited "self-initiated" cessation; that is, they have appeared to quit on their own without involvement in a formal quit-effort. By examining other variables measured at baseline, one can uncover predictors of later quitting (versus not quitting). A total of 17 such studies were found in the literature. Based on the results of these two types of studies – the formalized program and survey studies – suggestions for future research and practice directions are made.

A total of 11 tables are included in the review. The first six tables provide all raw data points used to construct the program study summary. Identification of investigators, years the work was conducted, data on methodological design, program contents, the target population, recruitment, retention, and follow-up, and data on quitting and percentage reduction in tobacco use are described in these tables. The next three tables summarize these data as a function of program theory and modality, and rank programs on outcomes and methods. The last two tables show the methods and target populations for the self-initiated quit studies, and summarize the results of each of these studies.

### Sixty-Six Cessation Intervention Studies

#### Selection of Studies

Among persons in the United States who have ever smoked daily, 16% have tried a cigarette, and 2% began smoking daily, by 12 years of age. Further, 82% have tried a cigarette, and 53% began smoking daily, by 18 years of age; and 98% have tried a cigarette, and 95% began smoking daily, by 25 years of age [2], p. 65]. Since most youth begin daily smoking by 25 years of age, the cessation studies included here generally targeted tobacco-using youth between the ages of 12 and 25 years old. However, seven studies were included that encompassed ages outside this range to allow the review to be as inclusive as possible. Three studies with wide age ranges included somewhat younger youth. Librett [13] included a through-study age range of 11–18; Patten et al. [14] included a through-study age range of 11–17; and Popham et al. [15] included a through-study age range of nine to 18 years. Also, four studies with wide age ranges included somewhat older adults. Etter, Ronchi, & Perneger [16] included a through-study age range of 24–33; Glasgow et al. [17] included a through-study age range of 15–35; Quinlan & McCaul [18] included a through-study age range of 18–55; and Zavela, Harrson, & Owens [19] included a through-study age range of 18–39.

The studies selected aimed to stop or reduce tobacco use among teens and young adults and included at least some data regarding contents of the cessation effort and attempts at quitting. Some of these programs targeted other age groups as well (e.g., older adults), but only data on teens and young adults were examined. Middle-school-based prevention programs were excluded from the present analysis because they did not supply any cessation information.

The contents of these programs could involve use of any cessation theory (e.g., social influence, motivation enhancement) and type of community unit to induce change (e.g., mass media, family, policy, or school). Thus, numerous theories and modalities of programming were included. All of these theories or modalities of program delivery were referred to as "programs", "Theory" refers to the theoretical content of the program. For example, an access reduction program, smoke-free areas, or taxes on cigarettes would be referred to as representatives of "supply reduction" theory, since they ultimately aim at making tobacco more difficult to obtain or use as a means to try to reduce tobacco use behavior. "Modality" refers to the community unit within which the cessation program is implemented. For example, use of a smoke-free area supply reduction approach (the theory) could be completed within one building, all public buildings in a city, or all public buildings throughout a state or country (different modalities).

The present 66 studies were compiled by referencing five different sources. Nineteen studies were included from a previous review on the subject [12]. Seventeen studies were presented as cessation programs in that earlier review. Two studies from that review were senior high school-based prevention programs, but they were included in the present review because these programs targeted a sizable number of baseline tobacco users and introduced some quit information. Seven more cessation studies were found in a review developed by Health Canada [20,21]. Twenty-six studies were found by engaging in a search of PsycINFO and MedINFO from 1970 to January, 2001. A subject search was performed using the phrases "adolescent tobacco", "tobacco cessation", "adolescent tobacco cessation" and "teen tobacco cessation". The references of all articles found were also searched but no additional articles were found. One study came from searching the World Wide Web, using the Google search engine [22]. Finally, 13 studies were found through word-of-mouth (from colleagues currently engaged in adolescent tobacco cessation work).

These five sources generated 66 tobacco-use cessation studies that probably are all the published or statistically evaluated cessation programs between 1975 and January 2001. Fifty studies were conducted in the United States, and 16 studies were conducted in countries outside of the U.S (five in Canada, three in the UK, two in Australia, and one each in China, Finland, New Zealand, Norway, Sweden, and Switzerland). Of these 66 studies, 47 had been or are going to be published in peer review journals. Six of these 66 studies were conducted in the 1970s, 15 were conducted in the 1980s, 43 were conducted in the 1990s, and two had been conducted in 2000–2001. Thus, teen tobacco use cessation research seems to have become a more active research arena beginning in the 1990s. Table 1 presents the 66 studies selected including investigators, project name, project site, year data was collected, and where the data was reported (see Table 1).

**Table 1:** Cessation studies identification

Investigators	Project name/site	Years done	Where reported?
Ary et al.	Project PATH, Oregon Research Institute, OR	1988–1989	JBM, 1990
Aveyard et al.	Univ. of Birmingham, West Midlands, UK	1997–1998	BMJ, 1999
Baskerville, Hotte, Dunkley	Univ. of Ottawa, CAN	~1993	Health Canada, 1997
Bauman et al.	Univ. of NC-Chapel Hill, U.S.-wide sample; Family Matters	1996–1999	Prevention Science, 2000
Beaglehole et al.	Wellington, New Zealand	1976	New Zealand Medical J, 1978
Biener et al.	Univ. of Massachusetts, Boston, MA, State-wide	1993–1994	AJPH, 1998
Chakravorty	PRC, UNIV.IC, IL	1991	DAI, 1992
Charlton	Univ. of Manchester, UK	~1988	HER, 1992
Cinnomin, Sussman	Conejo Valley HS, CA	1992	Book, 1995
Colby et al.	Boston Univ., VAMC-P, H&RIH	~1997	JCCP, 1998
Coleman-Wallace et al.	Emory Univ., GA LL, CA, schools in CA	1996–1998	J School Health, 1999
Corby et al.	Wayne St. Univ., Detroit, MI	~1999	Exp Clin Psychopharm, 2000
Digiusto	New S Wales D.H., AUST	1989	Book, 1994
Dino et al.	ALA-FL, WV Univ.	1999	Manuscript under review
Eakin, Severson, Glasgow	Oregon Research Institute, OR	1986	NCI Monographs, 1989
Etter, Ronchi, Perneger	Univ. of Geneva, Geneva, Switzerland	1996	J Epid Comm Health, 1999
Fibkins	S-WRHS, NY	1990	NASSB, 1993
Forster et al.	Univ. of Minnesota,14 MN communities	1993–1996	AJPH, 1998
Glasgow et al.	Oregon Research Institute, OR	~1997	JCCP, 1999
Glover	EC Univ., NC	1985	AJPH, 1986
Goldberg, Gorn	McGill Univ., Quebec, CAN	~1981	J Communication, 1982
Greenberg, Deputat	St. Univ. of NY, NY	1975	JSH, 1978
Hafstad, Aaro, Langmark	Univ. of Bergen, Buskerud County, Norway	1992	HER, 1996
Horn et al.	ALA, WVUNIV.	1998	HE, 1999
Horswell, Horton	Ottawa, CAN	~1996	Health Canada, 1997
Hotte et al.	Ottawa, CAN	1997	Health Canada, 1997
Hurt et al.	NDC, Mayo, Rochester MN and LaCrosse, WS	1997	Arch Ped & Adol Med, 2000
Jason, Mollica, Ferrone	DePaul, Chicago, IL	1978	PM, 1982
Jerome	Reston, VA	~1997	, 1998
Johnson et al.	HASP, IPR, USC, LA, CA	1981–1983	JBM, 1986
Kempf, Stanley	Rutgers Univ., Substance Abuse Treatment Campus, NJ	1994–1995	J Addictive Diseases, 1996
Killen et al.	CRDP, Stanford, CA	1986	JAMA, 1988
Lampkin	AMA, 5 School-based health centers (CO, DE, MI)	1997	AMA Technical Report, 1998
Librett	End Nicotine Dependence, Salt Lake City, UT	1998–2000	Unpublished doctoral dissertation, 2001
Lotecka, McWhinney	Oceanside, CA	~1981	IJA, 1983
Matson-Koffman, Miller	Atlanta, GA	1994	Conference presentation, 1995
McDonald, Roberts, Deeschaemaker	TCDC, Oakland, CA	1994	J Sub Abuse Tr, 2000
Mills, Ewy, Dizon	ACS-MN	~1977	HE, 1978
Murray, Prokhorov, Harty	Univ. of MN, State-wide campaign, MN and WS	1986–1990	PM, 1994
Myers, Brown	Univ. of SD, VAMC-SD, CA	1986–1989	Pediatrics, 1994
Myers, Brown, Kelly	Univ. of SD, VAMC-SD, CA, outpatients	~1997–1999	J Child Adol Substance Abuse, 2000
Pallonen	CPRC-Univ. of RI, RI	1991–1994	Tobacco Cessation for Youth Conference, 1996; Substance Use & Misuse, 1998
Patten et al.	NDC, Mayo, catchment areas in MN	1988–1997	Unpublished data, 2001
Patterson	Mishawaka, IN	~1983	The School Counselor, 1984
Pendell	C&I, Inc, MN	1995–1997	Manual material and handout at CDC, 1997; Pendell, 1996
Perry et al.	SHDP-Univ. of CA SF, Univ. of CA LA, CA	1978	AJPH, 1980
Perry et al.	HSPP, SU-ALA, CA	1980	Adolescence, 1983
Peters	Ottawa, CAN	~1993	Health Canada, 1997
Peterson, Clark	Melbourne, AUST	~1985	Psych Rep, 1986
Popham et al.	Univ. of CA LA, CA State-wide media campaign	1990–1991	Am J Prev Med, 1994
Prince	Project Tobacco, No Thanks! GGI, Ventura County, CA	~1993	Adolescence, 1995
Quinlan, McCaul	ND St. Univ.-Fargo	~1998	Health Psych, 2000
Rigotti et al.	TRTC, Harvard, 6 MA communities	1994–1996	NEJM, 1997
Skjoldebrand, Gahnberg	Public Dental Service, Uppsala Sweden	1990–1993	Swed Dent J, 1997
Smith et al.	Mayo Clinic NRC, Rochester, MN	1993–1995	Pediatrics, 1996
St. Pierre, Shute, Jaycox	~HS in CA	1982	HE, 1983
Suedfeld et al.	Rutgers, NJ undergraduates	~1971	IJA, 1972
Sussman, Burton et al.	Project TNT, IPR, PRC, CA, IL	1990	Book, 1995
Sussman, Dent, Lichtman	Project EX IPR, CA	1998	Addict Beh, 2001
Sussman, Dent, Stacy	Project TND IPR, CA	1997–1998	AJHB, in press
Townsend et al.	MRC-E&MCU, Middlesex, GB	1990	British Medical Journal, 1991
Vartiainen et al.	Helsinki, Finland	~1997–1999	Under review, 2001
Wakefield et al.	Univ. of IL at Chicago, IL; U.S. Nation-wide survey	1996	British Medical Journal, 2000
Weissman et al.	Oregon Research Institute, OR	~1985	Psych Add Beh, 1987
Zavela, Harrison, Owens	UNCT, Greeley, CO	1990	APHA Meeting Presentation, 1991
Zheng	USC, CA-pilot data collected in Wuhan, China, Project EX	2000	Unpublished data, 2000

NA = not applicable; NR = not reported; for specifics on "Where reported?" see references section; ~ = approximately.

This review differs from most previous reviews that are typically completed in the arena of adolescent tobacco use prevention or cessation. Most previous reviews limited their selections to relatively rigorously evaluated studies consisting of at least a quasi-experimental design, which includes a program group comparison to a control group [2,5,23-25]. Use of a comparison group permits a calculation of relative quit-rates (program minus control). Single-group studies assess simply how many people quit in a particular treated group without comparison to a control [26]. These were included in the present review to increase the study sample size (considering the state of the science in this arena), and because in some cases use of a control group was not possible (e.g., in some of the policy-type studies).

A gross comparison measure was calculated by pooling control group estimates across the quasi-experimental and experimental studies. In addition, all studies that addressed teen cessation are included herein; from education-program efforts to policy or mass media efforts. Including single-group studies raised the total number of studies reviewed from 37 (15 experimental and 22 quasi-experimental) to 66 (see Table 2).

**Table 2:** Cessation studies – methodological design

Investigators	Methodological design	Biochemical validation?
Ary et al.	Experimental – two condition: multigrade level (6th through 11th) social influence prevention, standard care; also parent messages randomly assigned to 12 schools within program condition	Yes
Aveyard et al.	Experimental – two condition: expert system and three class sessions based on transtheoretical model, standard health education (a little to motivate quitting)	No
Baskerville, Hotte, Dunkley	Quasi-experimental – quit-and-win contest and smoke free month, standard care control	Yes
Bauman et al.	Experimental – family program, standard care control	No
Beaglehole et al.	Quasi-experimental – classroom program, standard care control	No
Biener et al.	Single-group – random digit dialing	No
Chakravorty	Experimental – three condition: mintsnuff, chewing gum control, lecture only	Yes
Charlton	Quasi-experimental – pilot clinic ("courses"), self-help	Yes
Cinnomin, Sussman	Experimental – two condition: social influence/stress-coping, chemical addiction	Yes
Colby et al.	Experimental – two condition: motivational interview, brief advice	Yes
Coleman-Wallace et al.	Quasi-experimental – three condition: Tobacco Education Program (TEG) for precontemplators, Tobacco Awareness Program (TAP) for those who want to quit, control; 57% mandatory-punish (in TEG)	Yes
Corby et al.	Single-group – within subject replicated ABA design, 1 week each with a two week follow-up	Yes
Digiusto	Quasi-experimental – three condition: lunchtime quit clinic, class-time quit clinic, standard care control	Yes
Dino et al.	Quasi-experimental – two condition: not on tobacco (NOT), brief intervention	Yes
Eakin, Severson, Glasgow	Single-group – within subject replicated AB design	Yes
Etter, Ronchi, Perneger	Quasi-experimental – two condition: smoke-free program-four buildings/limited areas/cessation counseling service, control (other buildings)	No
Fibkins	Single-group – 1 group clinic	No
Forster et al.	Experimental – two condition: policy program, standard care control	No
Glasgow et al.	Experimental – two condition: brief intervention, simple advice to quit smoking	Yes
Glover	Single-group – two pilot clinics	Yes
Goldberg, Gorn	Quasi-experimental – two condition: personal involvement, standard care control	No, did use behavioral observation
Greenberg, Deputat	Quasi-experimental – four condition: fear, facts, values, standard care control	No
Hafstad, Aaro, Langmark	Single-group – mass media campaign for teens	No
Horn et al.	Quasi-experimental – two condition: not on tobacco (NOT), brief intervention	Yes
Horswell, Horton	Quasi-experimental – peer led school clinic, standard care control	No
Hotte et al.	Quasi-experimental – quit 4 life small groups plus kit, quit 4 life self-help kit-only	No
Hurt et al.	Single-group – nicotine patch therapy	Yes
Jason, Mollica, Ferrone	Quasi-experimental – 3 condition: role-play plus discussion, discussion-only, control	Yes
Jerome	Single-group – Life Sign computer assisted	Yes
Johnson et al.	Quasi-experimental – 4 condition: social curriculum/familiar media role models, social curriculum/unfamiliar media role models, health curriculum/familiar media role models, health curriculum/unfamiliar media role models	Yes
Kempf, Stanley	Quasi-experimental – 2 condition: smoke-free policy, standard care control	No
Killen et al.	Quasi-experimental – 2 condition: special intervention, standard care control	Yes
Lampkin	Single-group – pretest-posttest (averaged follow-up)	No
Librett	Single-group – pretest-posttest	No
Lotecka, McWhinney	Quasi-experimental – two condition: matched groups: coping, information	No
Matson-Koffman, Miller	Single-group – quit and win/tobacco free teens, school clinic	NR
McDonald, Roberts, Deeschaemaker	Single-group – consecutive cohorts	No
Mills, Ewy, Dizon	Single-group – two cohorts, senior high and junior high	No
Murray, Prokhorov, Harty	Quasi-experimental – two condition: statewide anti-smoking campaign in Minnesota, Wisconsin as control; sequential 9th grade cohorts	No
Myers, Brown	Single-group – consecutive cohorts	No
Myers, Brown, Kelly	Single-group – consecutive cohorts at three facilities	Yes
Pallonen	Single-group – feasibility study	Yes
Patten et al.	Single-group – retrospective cohort study	Yes, at baseline only
Patterson	Single-group – feasibility study	No
Pendell	Single-group – consecutive cohorts	No
Perry et al.	Quasi-experimental – two condition: special intervention, standard care control	Yes
Perry et al.	Experimental – three condition: long-term health effects, social consequences, physiological effects; also two teaching modalities (teacher, college student)	Yes
Peters	Single-group – quit 4 life self-help kit requesters	No
Peterson, Clark	Quasi-experimental – two condition: discussion group, standard care control group	No
Popham et al.	Single-group – state-wide: looks at those exposed and not exposed to campaign	No
Prince	Quasi-experimental – three condition: peer led, adult led, standard care control group	No
Quinlan, McCaul	Experimental – three condition: stage-matched (to precontemplation stages of change), stage-mismatched (action material offered), assessment only	No
Rigotti et al.	Quasi-experimental – two condition: enforcement or non-enforcement of tobacco sales laws	No
Skjoldebrand, Gahnberg	Single-group – all teens who came to the clinic for check-ups	No
Smith et al.	Single-group – non-randomized open label trial	Yes
St. Pierre, Shute, Jaycox	Single-group-group clinic pilot of ACS I-Quit	No
Suedfeld et al.	Experimental – four condition: use of sensory deprivation (Senory D) chamber or not, with a tobacco use health consequences message or not	No
Sussman, Burton et al.	Experimental – three condition: psychosocial dependency, chemical addiction, wait list control	Yes
Sussman, Dent, Lichtman	Experimental – three condition: clinic plus school-as-community, clinic only, standard care control	Yes
Sussman, Dent, Stacy	Experimental – three condition: health educator led classroom, self-instruction, standard care control	Yes
Townsend et al.	Single-group – "1-shot"	No
Vartiainen et al.	Single-group-quit-and win approach	Yes
Wakefield et al.	Nation-wide survey of the extent of smoking restrictions on teen smoking	No
Weisman et al.	Single-group-AB design	Yes
Zavela, Harrison, Owens	Experimental – three condition: mint snuff, bubble gum, comparison (no oral substitute lecture-only group)	No
Zheng	Single-group – quit clinic pilot	Yes

NR = not reported.

#### Program Components

Tobacco Product Focus. One study presented data on both cigarette smoking and smokeless tobacco use [27]. Four studies pertain only to smokeless tobacco use [19,28-30]. The remaining studies address only cigarette smoking cessation.

##### Theoretical Contents

The theoretical contents of the 66-cessation studies selected were derived from several types of behavior change theoretical frameworks. It is a difficult task to try to delineate different theoretical structures since some programs use only small "pieces" of theories and others show both overlap and non-overlap of multiple theoretical contents. However, programs were grouped together that shared a similar general theoretical perception of teen cessation. Different groups of studies, while sharing some minimal overlapping features, reflect distinct approaches. The grouping produced seems reasonable; however, future work might consider grouping studies through use of multiple reviewers.

Collapsed across different modalities of programming, a total of eight types of theoretical foci seemed reflected in these studies:

1. Social influence-oriented: to combat social influences that serve to promote or maintain teen tobacco use.

2. Cognitive-behavioral: instruction in cognitive-behavioral self-monitoring and coping skills to quit and maintain tobacco use cessation (e.g., smoking diary, stress coping).

3. Motivation enhancement: techniques to clarify desire for change and reduce ambivalence toward change. This may include, but is not restricted to, a specific strategy such as motivational interviewing.

4. Response-contingent reinforcement: reinforce quit-behavior with the chance for extrinsic rewards such as money or prizes.

5. Supply reduction: arrange the social environment such that tobacco is more difficult to obtain or use (e.g., price increases or restricted access).

6. Addiction/recovery-derived: use of means to ease physical effects of withdrawal, or emphasis on recovery from addiction.

7. Stages-of-change: techniques directly derived from the well-known model of change (e.g., tailored cost and benefit information, treating contemplation to quit and quit strategies as involving distinctly different processes of change).

8. Affect clarification: techniques to clarify and remove conflicted affect, and thereby permit pursuit of health including tobacco use cessation.

Eleven studies attempted to counteract social influences to use tobacco [15,31-40]. In these studies, the key information provided to help youth quit smoking was focused on combating social influences that may maintain smoking behavior. Such information included refusal assertion skill instruction, instruction in awareness of tobacco industry promotions, media and peer social influences, and correction of social informational inaccuracies. While sharing this common focus on counteraction of social influences that may maintain tobacco use, some of these programs also contained notable unique features. The Beaglehole et al. study [32], for example, addressed physical consequence information as well as social influence information using the health belief model as a teaching guide. Killen and colleagues [36] focused on the social attractiveness of exercise and healthy food intake, as well as on smoking behavior. Peterson & Clark [39] focused on instruction in social influences and smoking, and also placed a focus on group decision-making to cut down on smoking levels. Finally, Townsend and colleagues [40] focused on normative social influence (i.e., conformity to achieve acceptance) by teaching skills such as refusal assertion, rather than by attempting to counteract more covert social informational influences that aim to achieve attitudinal similarity (e.g., counteracting media influence). Conducted in a medical office, this study also addressed physical consequences information and general health practices [40].

Sixteen studies [8,13,14,23,27,29,30,41-50] focused on instruction of cognitive-behavioral coping techniques. In particular, they focused on uncovering the topography of one's tobacco use (e.g., reasons for smoking and quitting, self-monitoring) and how to cope effectively with stress (e.g., seek out social support, relaxation, wait out urges, self-management, problem solving). While most of these programs discussed at least briefly counteraction of social influences and chemical dependence (e.g., coping with withdrawal symptoms), the emphasis was on intra-personal coping. One unique aspect here is that two studies grouped coping with different functions of tobacco use in separate conditions [27,42]. For example, Sussman et al. [27] compared coping with psychosocial dependency on tobacco separately from coping with chemical addiction to tobacco. Eakin and colleagues [29] introduced a pneumonic device, the "Four A's": Avoid, Alter, Alternatives, and Activities, as a strategy for coping with the difficulty of cessation.

Nine studies emphasized motivation enhancement [17,51-59]. These studies dedicate a significant percentage of the program to making an attempt to increase smokers' motivation to quit smoking prior to providing instruction in life skills, social influences, and/or chemical dependence. Motivation enhancement helps participants to clarify their direction of change and increases their willingness to change. Motivation enhancement may include such strategies as giving advice, removing cognitive impediments to change, providing choices, and reconciling discrepancies between current behavior and desired goals [60,61]. Within this set of studies, two emphasized a single type of motivation manipulation – the notion of establishing cognitive consistency. Specifically, Goldberg & Gorn [54] made use of the concept of personal involvement to try to get college-age smokers to quit by serving as mentors of younger youth, as part of a tobacco education/communications class. Hafstad, Aaro & Langmark [55] contrasted popular opinions and realities of being a smoker as part of a mass media program.

Five studies emphasized principles of response-contingent reinforcement [62-66]. The main goal was to test whether an offer of an extrinsic reinforcement or the possibility of reinforcement to participants would decrease the frequency of tobacco use behavior [61,67]. Among this set of studies, three made use of a Quit-and-Win Contest concept combined with a primarily a chemical addiction orientation [62,64,65]. Two studies were contingency-based [63,66]. Corby and colleagues [63] developed a very brief contingency-based study to learn about the effects of contingencies on initial cessation; Weissman et al. [66] designed a contingency focused study with a 15–20-session clinic program that rewarded adequate participation with money.

Seven studies took primarily a supply reduction approach. Supply reduction approaches aim to arrange the environment in such a way that tobacco is more difficult to obtain or use [68]. By making tobacco harder to obtain or use, it is theorized that costs (financial, time, or social) of tobacco use increase for users and that they may have more reason to think about quitting. In addition, supply reduction approaches provide a large social environmental statement of disapproval regarding tobacco use. There are at least three types of supply reduction approaches. One approach is taxation to raise the price of tobacco [69,71]. One may expect that a percentage increase in the price of tobacco will result in a corresponding increase in quit-rates. A second supply reduction approach is the establishment of smoke-free areas to limit where tobacco can be smoked [16,70]. A third approach consists of limitations on where and by whom tobacco can be purchased or obtained [72-74], reducing overall access to tobacco products. One unique study, Wakefield et al., [74] examined the effects of access reduction across multiple contexts. Another one of these studies also included social influence education, in addition to use of multiple supply reduction approaches [71].

Nine studies took a primarily addiction/recovery approach [19,22,28,75-80]. These programs emphasized strategies to ease the physical effects of withdrawal/addiction (use of pharmacological adjuncts or substitutes) or emphasized recovery from physical addiction.

Smith et al. [80] and Hurt et al. [76] studied the provision of nicotine patches to teens, and Hurt made use of a physician-assisted model developed for adults (the 4-A's, ask, advice, assist, arrange follow-up). Jerome's [22] study involved the use of a hand-held computer to facilitate gradual withdrawal from nicotine (LifeSign) combined with attendance at weekly support groups. Three studies focused on providing support groups modeled on twelve-step programs [75,77,78]. Two studies, Chakravorty [28] and Zavela, Harrison, and Owens [19], were substitution-based studies. Both studies were developed for smokeless tobacco users, and they substituted tobacco use with use of a non-tobacco, crushed mint leaves product (Mint Snuff, Oregon Mint Snuff Co., 1–800-EAT-MINT). A final study involved screening in a dental clinic, provision of dental professional advice, and referral to a nicotine detoxification program [79].

Seven studies [7,18,81-85] were Transtheoretical (stages of change) model-based. In this model program material is framed for the participants stage of change, to help facilitate the subjects' movement through the quitting process [also see [86,87]]. These stages are: 1) precontemplation, during which a smoker has not considered quitting; 2) contemplation, during which a smoker is thinking about quitting; 3) preparation, during which a smoker is prepared to quit; 4) action, during which a smoker is actively involved in quitting; and 5) maintenance, during which a smoker has quit and is trying to stay off tobacco. Program contents were tailored to the appropriate stages of change of participants. Early in the stages of change, subjects' costs minus benefits of smoking are calculated (e.g., financial, social costs versus withdrawal reduction benefits), and self-reevaluation and insight are obtained (the subject decides to quit when perceived costs are reliably greater than benefits). Later in the stages of change, various skill-building and quitting behaviors are learned. Pendell's work [82,85] includes two different programs, the Tobacco Education Group (TEG) and the Tobacco Awareness Program (TAP). The TEG program addresses specifically adolescents at the precontemplative or contemplative stage, while the TAP program addresses specifically those students at the preparation, action, and maintenance stages. Quinlan and McCaul [18] made use of stage of change concepts within a brief university-based quit-clinic. Mills and colleagues [83] made use of social influence and buddy-contracting concepts, as part of later stages of change action steps. Lampkin's [7] study made use of a brief motivation enhancement interview, although it focused on stages of change concepts. Aveyard et al. [81] and Pallonen's [84,86,88] programs were computer-based and provided relatively specific tailored feedback depending on the participant's stage of change.

Finally, two studies involved an affective education-type approach. These are based on the premise that through clarification or release of conflicted – or pent-up – affect, the participant returns to a healthy affective state which would subsequently lead to elimination of unhealthy behavior such as smoking. Greenburg and Deputat's [89] study focused on an affective education/values clarification model, comparing it to information and fear approaches. Suedfeld et al. [90] involved use of a sensory deprivation chamber to "unfreeze" attitudes and permit smoking cessation.

##### Modality of Programming

Modality refers to the channels or contexts within which the cessation programming is offered. Seven modalities of programming were delineated. These were school-based clinics, medical or recovery clinics, system-wide efforts, classroom-based, computer-based, family-based, and use of a sensory deprivation chamber.

In twenty-eight studies programming was delivered in a school-based clinic, the most popular modality (43%). School-based clinics involve the implementation of highly interactive, private sessions for small groups of youth developed specifically for tobacco use cessation, on the grounds of schools. These clinics are composed of students from the school, but are delivered outside of the regular classroom context. Generally, five to 15 youths will participate in each clinic group in an empty classroom or office, and groups often will meet during school hours. Frequently, youth are released from class to attend the clinic.

Another 13 studies involved a medical or recovery clinic. These efforts often are similar to school-based clinics but involve attendance at a medical or recovery facility. In some cases, youth are treated individually in addition to or in place of within a group context.

Another 11 studies involved system-wide efforts (e.g., mass media campaigns, policy, or statewide). These efforts involve delivery of programming to complete or multiple social units (i.e., applies to everyone in a building, set of buildings, community, or even larger units such as states). As such, programming is delivered to very large numbers of people, and may involve different combinations of modalities (e.g., schools, the mass media, local business, and city leaders). Such programming may include various demand reduction and/or supply reduction components [68]. Thus, while the theory in operation may vary across different system-wide studies, the resulting program contents are delivered widely.

A total of 9 studies were classroom-based. This programming was delivered within intact classrooms as part of a classroom course. Three additional studies employed the use of computers (i.e., "expert systems" – iterative feedback from experts within a computer modality, hand-held or PC), primarily as a means of delivering a self-help program. While sometimes placed in a school setting computer lab, youth could use the computer tailored to their level of tobacco use and interest in quitting. In this case, the programming is tailored to the individual, who can set up his/her own time to use the material. One study was a family-based intervention [51], which involved self-help and telephone counseling to parents and their 12-to-14 year old children (focused on the home setting). Finally, one study [90] involved use of a sensory deprivation chamber at a university lab (see Table 3).

**Table 3:** Cessation studies – program contents

Investigators	Theoretical guide	Modalities	#Sessions
Ary et al.	Social influence-oriented prevention	Classroom program, three parent messages, videos	10
Aveyard et al.	Stages of change model – normative/ipsative feedback, smoking pros/cons; control-information on health	Three computer expert system lessons and three classes	6
Baskerville, Hotte, Dunkley	Contingency-based, quit-and-win contest contingent on smoke free month, buddy support, chemical addiction	System-wide, school-based contest and self-help materials, youth signed up from 10 high schools	NA
Bauman et al.	Motivation enhancement, parenting, modeling, social influences	Family-directed, through mail and phone calls	NA (5 booklets, 15 activities)
Beaglehole et al.	Social influence and health belief model versus standard care fear information and discussion	Classroom program with films	NR (semester course)
Biener et al.	Supply reduction: $0.25 excise tax	System-wide, state-wide reaction to price increase	NA
Chakravorty	Substitution (chemical addiction) and education	School clinic	2
Charlton	Cognitive-behavioral – "Packing it in?"	School clinic or self-help	6
Cinnomin, Sussman	Cognitive-behavioral, social influences, chemical addiction	School clinic	6
Colby et al.	Brief motivation enhancement-oriented	Medical hospital clinic	1
Coleman-Wallace et al.	Stages of change – TEG and TAP; cognitive-behavioral	Mostly school clinic, videos, cooperative learning	8
Corby et al.	Contingency-based, 1-week contingency management ($40)	CO measurement at a medical-type clinic	NA
Digiusto	Cognitive-behavioral, social influences	School clinic	6
Dino et al.	Cognitive-behavioral, social influences, chemical addiction	School clinic; single-gender groups led by same-gender facilitator; brief intervention – 20 minute quit advice and self-help material	12
Eakin, Severson, Glasgow	Cognitive-behavioral, coping skills – 4 As	At Oregon Research Institute, school-like clinic, small group meetings, 2–3 counselors, videos	3
Etter, Ronchi, Perneger	Supply reduction, policy, information campaign, self-help	System-wide, smoke-free areas, posters, leaflets, self-help quit manuals at part of university	NA
Fibkins	Recovery/addiction, student assistance counseling model, recovery concepts: "Five Hour a Day"	School clinic with counselor and nurse	6
Forster et al.	Supply reduction; make youth tobacco access a salient issue, change ordinances, change retailer and parent practices, enforcement of sale laws	System-wide, community organizer teams, group presentations, letter and petition drives, meetings with community leaders and retailers, media campaigns, purchase attempts	NA
Glasgow et al.	Brief motivation enhanced-oriented and follow-up support phone calls	Medical-like planned parenthood clinic, short video, brief counseling, 1–3 phone calls	1–2
Glover	Cognitive-behavioral-ACS Fresh Start	College/school clinic	2
Goldberg, Gorn	Motivation enhancement, personal involvement to help younger youth	College classroom program, films, advertisement analysis, social influence texts, discussion	~16
Greenberg, Deputat	Affect-oriented, fears, facts or values	School clinic, films	7
Hafstad, Aaro, Langmark	Motivation enhancement, cognitive consistency – popular opinions and being a smoker	System-wide, newspaper ads, poster, 1 TV and cin ema spot; home-mailed questionnaires	NA
Horn et al.	Cognitive-behavioral, social influences, chemical addiction	School clinic; single-gender groups led by same-gender facilitator; brief intervention – 20 minute quit advice and self-help material	14
Horswell, Horton	Social influence – Canadian Cancer Society's Fresh Start Program	School clinic: "Pack in Those Smokes"	3
Hotte et al.	Cognitive behavioral-consequences, coping with withdrawal	School clinic: "Quit 4 life"	7
Hurt et al.	Chemical addiction, brief 4A's intervention: advice to quit, self-help material	Medical clinic; nicotine patch use	7 (1 advice 6 patch checks
Jason, Mollica, Ferrone	Social influence, immediate and long-term consequences of smoking	Classroom program	6
Jerome	Chemical addiction, gradual with-drawal, self-help cognitive-behavioral guide	Hand-held computer, weekly support meetings at high school	8
Johnson et al.	Social influence and short-term consequences/long-term consequences; public commitment in both conditions	Classroom program, videos	4
Kempf, Stanley	Supply reduction, smoke-free policy versus no regulation of smoking outside the building	More a medical clinic context, residential therapeutic community drug treatment programs	NA
Killen et al.	Social influence, social-cognitive/increase attractiveness of 4 health practices	Classroom program	20
Lampkin	Stage of change, some addiction and mo tivation enhancement	School based health center (medical context)	4
Librett	Cognitive-behavioral, alter tobacco use expectancies, build self-efficacy	School clinic	6
Lotecka, McWhinney	Cognitive-behavioral, coping	School clinic	4
Matson-Koffman, Miller	Contingency-based, quit and win/tobacco free teens, contest, chemical addiction, phone counseling	School clinic	8
McDonald, Roberts, Deeschaemaker	Therapeutic community and "I quit" derived, adult cessation & recovery concepts	Medical, inpatient treatment	5
Mills, Ewy, Dizon	Stages of change-pre-contemplation, social influence, contract with buddy control	School clinic	8
Murray, Prokhorov, Harty	Social influences-oriented	System-wide, higher taxes on tobacco, school-based with smoke-free campuses and education, mass-media campaign on TV and radio and newspapers and billboards, and local community grants	NR
Myers, Brown	Recovery oriented	Medical, inpatient treatment – generalization to cigarettes	NA; 28 – not tobacco
Myers, Brown, Kelly	Motivation enhanced, with cognitive-behavioral and social influence material	Medical, outpatient treatment – at three facilities	6
Pallonen	Stages of change	Computer assisted	3
Patten	Cognitive-behavioral, chemical addiction, social influence	Medical clinic consultation, telephone and mail follow-up	2
Patterson	Cognitive behavioral-similar to f resh start	School clinic	6
Pendell	Stages of change – TEG and TAP	Mostly school clinic, videos, cooperative learning	16
Perry et al.	Comprehensive social influences	Prevention and cessation in classroom program, films, self-monitoring	4
Perry et al.	Comprehensive social influences, prevention component oriented	Prevention in senior high school classroom, films, self-monitoring	3
Peters	Cognitive behavioral-consequences, coping with withdrawal	System-wide, self-help quit kit "Quit 4 life", call a toll free number to request kit	NA
Peterson, Clark	Social influence, group decision to cut down	School clinic	3
Popham et al.	Social influence, health and social consequences, society's disapproval, profit motivation of tobacco industry	System-wide, TV, radio, outdoor and print media. 13 general audience ads, 2 youth-focused ads	NA
Prince	Cognitive behavioral	School clinic	6
Quinlan and McCaul	Stages of change-pre-contemplation (costs and benefits), action (quit techniques and quit date)	Brief university clinic-two activities and take home materials	1
Rigotti et al.	Supply reduction, enforcement of no-sales laws to minors	System-wide, health department distributed written information to vendors and penalized noncompliance; minors attempted to purchase tobacco from vendors, surveys	NA
Skjoldebrand, Gahnberg	Addiction model	Medical, 1 public dental clinic: interviews and advice about tobacco, waiting room posters, brochures, and video program, some instruction by dental nurses in a group-information format, referral to tobacco detoxification program offered	NA
Smith et al.	Addiction model	Medical clinic-like, patch, behavioral counseling with group support-coping, wellness, relapse prevention	8
St.Pierre, Shute, Jaycox	Cognitive-behavioral, I-quit, psychosocial dependency, chemical addiction	School clinic, video	6
Suedfeld et al.	Affect-oriented, SD to "unfreeze" attitudes and permit change	University lab; sensory deprivation (Sensory D) chamber	1 24 hour Sensory D session
Sussman, Burton et al.	Cognitive-behavioral, psychosocial dependency and chemical addiction	School clinic, videos	5
Sussman, Dent, Lichtman	Motivation enhancement, with social influences and chemical addiction material	School clinic, school meetings and events in 1 condition	8
Sussman, Dent, Stacy	Motivation enhancement-skills-decision making-chemical addiction	Continuation high school classroom program	12
Townsend et al.	Social influence	Medical, nurse or physician assisted, quit pamphlet, general practice setting	1
Vartiainen et al.	Contingency-based, quit-and-Win lottery (for ~$1,600)	System-wide, through schools, biochemically validated quitting to enter lottery	NA
Wakefield et al.	Supply reduction: restrictions on smoking at home, school and public places	System-wide, cross-sectional survey; self-reports	NA
Zavela, Harrison, Owens	Substitution (addiction), with coping-oriented material	College clinic	9
Zavela, Harrison, Owens	Substitution (addiction), with coping-oriented material	College clinic	9
Zheng	Motivation enhanced, with social influence and addiction	School-based clinic setup in community center	8

NA = not applicable; NR = not reported.

It should be mentioned that the division of programming by theory and modality is sound logically, however two theories tended to be associated with certain modalities of delivery. Social influence programming tended to be delivered in a classroom setting (in seven of 11 instances of its use) and cognitive-behavioral programming tended to be delivered in a school-based clinic setting (in 14 of 16 instances of its use). The other theories and modalities were more evenly crossed in design. Thus, interpretation of main effects findings on theory and modality needs to be tempered by this information.

##### Number of Sessions

Number of sessions was defined herein simply as the total number of meetings without respect to the length of the meetings or spacing of the meetings. Number of sessions is not an appropriate measure for 15 studies, which involved non-educational type programming such as supply reduction policy enforcement. However, amount of exposure to non-educational programming is an important factor and generally was of a year or more duration. Also, two educational-type programs failed to indicate number of sessions (see Table 3). Of the 49 programs that report number of sessions, the mean number of sessions was 6.7 (the mode was six sessions, 11 programs; then eight sessions, seven programs; then three sessions, five programs; then one or two sessions, four programs each), and the range was from one to 20 sessions. This is an important variable to measure because, for the educational-type of program, it is possible that number of sessions is related to program success. Specifically, the greater the number of sessions the more potent the effect may be. This type of relation is found among teens in drug abuse prevention programming [91] and among adults in tobacco use cessation programming [92]. The relation of number of sessions to program outcomes will be examined in the program outcomes section below.

#### Methodological Design

The most widely used methodological design was single-group. Twenty-nine single group studies evaluated cessation rates in a program group without comparison to a control; 22 quasi-experimental studies utilized a control group to compare naturally occurring cessation rates with those occurring in the program condition; and 15 studies utilized random assignment to conditions in order to maximize experimental validity. Reliance on single-group design was explained in one study by the difficulty encountered in recruiting enough tobacco users to create a control group [29]. Another study reported that the main purpose of the study was to test level of adolescent interest and willingness to continue participation regardless of actual cessation rates [88]. Thus, a control group of smokers may have been unwarranted considering the focus. Other studies used a single group design as a means of pilot testing a program for later work [e.g., [7]]. Still, other work examined community-wide efforts [e.g., [69]]. Finding an appropriate control group for community-wide efforts is very difficult and sometimes is not possible. In the case of such system-wide efforts, comparisons generally are made to nation-wide trends, based on deterioration of effects after termination of programming, or through replications in different locations. The Canadian Tobacco Control Research Initiative (CTCRI), when considering "best practice" programs, takes a widely shared stance that replications across quasi-experimental and case study designs are considered appropriate designs for evaluating community-based interventions [93]. The predominance of use of a single group design indicates a need for more rigorous research designs in adolescent tobacco cessation (see Table 2). However, wise use of quasi-experimental designs and replications of single group designs can permit inferences regarding relatively strong or weak programming [26].

##### Biochemical Validation

Three biochemical methods that have been used for over 10 years with adults and teens include exhaled carbon monoxide (CO), saliva thiocyanate (SCN), and saliva cotinine measurement [27]. Most teen cessation studies have used CO measurement perhaps because cost of analysis is the least expensive, even though it has a relatively brief half-life of three to five hours. Biochemical validation was reported in 30 studies (see Table 2). There is recent debate concerning applicability of biochemical validation to adolescent regular tobacco users considering that teens may metabolize nicotine differently (e.g., more quickly) than adults (suggested by Henningfield at CDC, 1997, personal communication). Further, some researchers suggest that use of this procedure may discourage participation in cessation programming [e.g., [46]], or may not be imperative if multiple self-report measures are used [e.g., [94]]. Still, approximately 15% of adolescents who report cessation are detected as still using tobacco when concurrent biochemical validation measures are collected [e.g., [27], collected saliva cotinine; [57], collected carbon monoxide (CO) breath samples]. Since biochemical validation may produce more accurate rates of cessation, this means of validation should be considered when reporting adolescent tobacco use cessation data.

#### Target Population

##### Number of Baseline Tobacco Users

Across all 66 studies, a mean of 659.5 adolescent or young adult tobacco users participated at baseline in the cessation studies (range = eight to 14,746). Tobacco use was defined for this statistic as the total number of users of any type of tobacco product, regardless how tobacco "use" was defined by the individual studies. The number of tobacco-using subjects at baseline was 90 or fewer subjects in 26 studies, was between 91 and 150 subjects in 13 studies, was between 151 and 400 subjects in 11 studies and was 401 or greater in 16 studies. Half the studies (n = 33) contained 120 or fewer subjects, two-thirds of the studies contained fewer than 211 subjects, and 75% of the studies contained fewer than 400 subjects (see Table 4). Of the 25 studies that contained more than 200 subjects, four were a classroom-based modality, one was expert system based, 11 were system-wide programs, eight were school-based clinics, and one was a medical-oriented clinic. Thus, all modalities except for family based and sensory deprivation (one study in each category) contained at least one study with a large sample size.

**Table 4:** Cessation studies – target population

Investigators	# Tobacco users at baseline	Minimal or mean level of tobacco use	Through study age range	% female	% white
Ary et al.	776	>1 cig./week	12–18	~50	89
Aveyard et al.	1090	>1 cig./week	13–14	50	86
Baskerville, Hotte, Dunkley	331	NR (quit – 30 day)	14–18	NR	NR
Bauman et al.	110	>1/30 days	12–15	57	85
Beaglehole et al.	128	>2 cpd	12–15	NR	NR
Biener et al.	216	>1/30 days	12–17	NR	NR
Chakravorty	83-ST	>1.5 dips/day	14–18	0	~95
Charlton	87	NR	16	~75	NR
Cinnomin, Sussman	60	16 cpd (quit – 7 day)	14–19	38	~30
Colby et al.	40	10 cpd (quit – 7 day)	14–17	58	65
Coleman-Wallace et al.	351	13 cpd	14–18	NR	NR
Corby et al.	8	19 cpd (quit – 7 day)	15–19	38	NR
Digiusto	~277	~12 cpd (quit – 7 day)	14–18	~50	NR
Dino et al.	346	~15 cpd (median quit – 8 day)	14–19	54	87
Eakin, Severson, Glasgow	25-ST	5–8 dips/day (quit – 7 day/1 slip)	14–18	0	NR
Etter, Ronchi, Perneger	582	11 cpd	~24–33	60	NR
Fibkins	27	NR	14–18	NR	NR
Forster	660	>1 cpd (quit – 7 day)	14–16	NR	NR
Glasgow et al.	506	12 cpd (quit – 30 day)	15–35	100	88
Glover	41-ST	NR (quit – 6 months)	18–22	0	NR
Goldberg, Gorn	141	Current, 100+life	18	41	NR
Greenberg, Deputat	100	.5 cpd	16–18	57	NR
Hafstad, Aaro, Langmark	497	>1 per week	14–15	65	NR
Horn et al.	163	~17 cpd	14–19	55	92
Horswell, Horton	36	NR	12–18	NR	NR
Hotte et al.	632	12 cpd	14–15	49	NR
Hurt et al.	101	~20 cpd (quit – 7 days)	13–17	41	95
Jason, Mollica, Ferrone	32	~4 cpd	14–16	~56	~35
Jerome	17	13 cpd (quit – 7 days)	15–17	47	82
Johnson et al.	448	Monthly smoking – 13%	16–18	NR	NR
Kempf, Stanley	132	14 cpd	13–17	18	28
Killen et al.	~180	Weekly smoking – 16%	14–16	45	69
Lampkin	121	9 cpd (quit – 30 days)	15–18	66	64
Librett	212	9 cpd	11–18	59	77
Lotecka, McWhinney	49	7 cpd (quit – 7 days)	14–18	NR	NR
Matson-Koffman, Miller	80	NR	14–18	NR	NR
McDonald, Roberts, Deeschaemaker	51	27 cpd (quit – 7 days)	12–19	46	NR
Mills, Ewy and Dizon	34	~14 cpd	12–18	NR	NR
Murray, Prokhorov, Harty	450	>1 per week	14–15	NR	NR
Myers, Brown	141 (last 3 months)	13 cpd (quit – 7 days)	12–18	40	79
Myers, Brown, Kelly	35	>1 per week	13–18	40	71
Pallonen	135	10 cpd (quit – 7 days)	16–18	54	90
Patten et al.	96	~17 cpd (quit – 7 days)	11–17	38	92
Patterson	21	NR (quit – 2 days)	14–18	NR	NR
Pendell	3955	NR	12–18	NR	NR
Perry et al.	243	>1 per month – 27%	16	54	NR
Perry et al.	82	>1 per week	16	~50	NR
Peters	635	>1 cpd (quit – 7 days)	15–19	NR	NR
Peterson, Clark	22	8 cpd	14–16	100	NR
Popham et al.	7000	>1 per month – 13%	9–18	53	50
Prince	110	12 cpd	16–18	46	NR
Quinlan and McCaul	94	~13 cpd	18–55; mean = 22 (SD = 7)	64	100
Rigotti et al.	~2,900	>1 cpd	13–17	52	76
Skjoldebrand, Gahnberg	101	>1 cpd	12–17	38	NR
Smith et al.	22	23 cpd (quit – 7 days)	13–17	68	NR
St. Pierre, Shute, Jaycox	12	NR	16–18	42	NR
Suedfeld et al.	40	18 cpd	19–22	0	NR
Sussman, Burton et al.	244	NR (quit – 7 days)	14–18	50	60
Sussman, Dent, Lichtman	335	8 cpd (quit – 30 days)	14–19	36	27
Sussman, Dent, Stacy	583	~8 cpd (quit – 30 days)	14–19	34	43
Townsend et al.	68	7 or more cpd	13–17	54	NR
Vartiainen et al.	3241	> 1 per month (~8 per month)	15–24	45	NR
Wakefield et al.	14,746	> 1 per month	14–17	54	47
Weissman et al.	11	18.5 cpd (quit – 7 days)	13–18	46	NR
Zavela, Harrison, Owens	42-ST	7.6 dips per day	18–39 (mean = 20.7)	3	NR
Zheng	46	5 cpd (quit – 7 day)	16–17	7	0

NA = not applicable; NR = not reported; cpd = cigarettes per day; ST = smokeless tobacco users; SD = standard deviation; ~ = approximately; > = greater than.

The fact that half of the studies included 120 or fewer participants, many involving two or more conditions in the design, highlights that much of the work completed in teen cessation research is completed with under-powered samples. (For example, to achieve an effect size = 0.2 with 80% power, in a simple two-group design, a sample size of approximately 190 would be needed in each group, 1-tailed, p < .05; or 380 subjects would be needed if the study was two-tailed).

##### Age

As mentioned previously, the target age for these adolescent cessation studies was between 12 and 25 years old. However, seven studies were included in which target ages were outside this range. These exceptions were made because most of the youth in these seven studies fell within the targeted 12–24 years old age range, and the review was designed to be as inclusive as possible. Thus, age in the study set ranged from nine to 55 years old. Collapsing age range across studies, the modal ages are as follows: 16 years old (54 studies), 15 (51 studies), 14 (46 studies), 17 (46 studies), 18 (40 studies), 13 (19 studies), 12 (13 studies) and 19 years old (13 studies). A total of three or fewer studies looked at people 11 years old or younger. Among the studies that included nine to 11 year olds (as well as older youth), one was a school-based clinic [13], one was a medical-based clinic [14], and one was a system-wide study (statewide campaign; [15]). A total of seven studies looked at people 20 years or older. The Etter, Ronchi, & Perneger [16], Glasgow et al. [17], Glover [30], Quinlan & McCaul [18], Suedfeld et al. [90], Vartiainen et al. [65], and Zavela, Harrison, & Owens [19] studies, which examined older youth and adults, primarily were college-based studies. Within-study ranges reflect the "through-study" age range or the youngest age of those who were participating at the beginning of the study and the oldest age of those who were participating at the end of the study (see Table 4).

##### Gender and Ethnicity

Gender of participants was not reported or could not be estimated in 15 studies. Females were a majority of the sample in 21 studies (41% of studies that reported gender). Ethnicity was not reported in 38 studies. Majority white ethnicity was reported in 20 studies. Thus, eight studies reported white participants as being a minority of those represented. It can be tentatively suggested that teen cessation research needs to provide more demographic specifics (see Table 4). Such specifics are needed to discern variation in strength of effects as a function of gender or ethnicity [e.g., [43]].

#### Recruitment

##### Means of Recruitment

Five studies failed to report any recruitment data, including means of recruitment (Table 5). Among those studies that did report means of recruitment, the most widely used form was person-to-person, which was employed in 16 studies. With person-to person recruitment, researchers utilized word of mouth to interest subjects in cessation clinics. Smokers were approached by researchers, staff, or other youth at lunch, in a smoking section, or at other locations (e.g., youth "hang-out" areas) during the day and were encouraged to bring friends or spread the word. The next most popular method, employed by 15 studies, offered monetary incentives or compensation (four of these studies made use of a monetary contest or lottery).

**Table 5:** Cessation studies – recruitment

Investigators	Means of recruitment	Reach (recruited/total tobacco users notified)	Retention (% at posttest/attended 1st session)	Follow-up (% at follow-up/completed pretest)
Ary et al.	1. classroom prevention program	92%	100%	76%
Aveyard et al.	1. use of whole classes as part of personal health and social education lessons	90%	NR	89%
Baskerville, Hotte, Dunkley	1. contest	13%	66%	NR
	2. home room class announcement			
Bauman et al.	1. telephone screening	55%	NA	73%
Beaglehole et al.	1. classroom education program	99%	NR	92%
Biener et al.	1. telephone screening based on random-digit-dialing	~75%	NA	NA
Chakravorty	1. person-to-person	NR	95%	NA
	2. PA announcement			
	3. flyer			
Charlton	1. class presentation	26% joined clinic	~33% of clinic attendees	39%
Cinnomin, Sussman	1. class presentation	55%		
	2. person-to-person		100%	85%
Colby et al.	1. patient assessment (screening) and information about project	85%	NR	95%
	2. money ($20)			
Coleman-Wallace et al.	1. school district support and announcements	21%	77%	NA
	2. money ($3) for control group			
	3. mandatory to avoid suspension (57% subjects)			
Corby et al.	1. newspaper ads	NR	100%	100%
	2. money ($135 total possible)			
	3. referrals from community agencies			
	4. person-to-person			
Digiusto	1. posters	21% (39% in class time, 11% in lunchtime)	80%	~80%
	2. assembly announcement			
	3. classroom announcement			
	4. class release time in 1 condition			
Dino et al.	1. poster "ads" placed in likely smoking areas and public areas around the school	~10%	65%	48%
	2. PA announcement			
	3. person-to-person			
	4. class release time			
Eakin, Severson, Glasgow	1. person-to-person	76% agreed to be in study/approached	84%	80%
	2. referrals			
	3. money ($60)			
Etter, Ronchi, Perneger	1. names of university administrative files (screening)			
	2. surveys by mail	77%	NA	83%
Fibkins	1. person-to-person	9%	100%	NA
	2. referrals to school counselor and nurse			
Forster et al.	1. classroom surveys (whole classes)	93%	NA	93%
	2. media campaigns			
	3. policy enactment and enforcement			
Glasgow et al.	1. chart review/screening (approach subject at contraceptive visit)	74% agreed to be in study/approached	NR	91%
	2. money ($70)			
Glover	1. mandatory	NR	100%	100%
Goldberg, Gorn	1. mandatory	NR	100%	~65%
Greenberg, Deputat	1. person-to-person	100% – stopped at first 100; perhaps 40% of tobacco users at school	95%	78%
	2. referrals			
	3. 2 unit credit for complete participation			
	4. mandatory			
	5. principal support			
Hafstad, Aaro, Langmark	1. county-wide mass media campaign	NR	NA	66%
	2. home-mailed questionnaire, with three reminders			
Horn et al.	1. poster "ads" placed in likely smoking areas and public areas around the school	~10%	72%	NA
	2. PA announcement			
	3. person-to-person			
	4. class release time			
Horswell, Horton	NR	NR	NR	NR
Hotte et al.	1. class credit	74%	46%	31%
	2. some type of school-wide announcements			
Hurt et al.	1. flyers in schools	NR	70%	57%
	2. press releases, TV and radio announcements,			
	3. telephone interview/screening			
	4. $100 compensation			
Jason, Mollica, Ferrone	1. classroom program	~100%	~100%	84%
Jerome	1. person-to-person	~23%	88%	NA
	2. referral by assistant principal			
Johnson et al.	1. classroom program	~100%	36%	17%
Kempf, Stanley	In-patient facility – NA	98%	NA	77%
Killen et al.	1. classroom program	~100%	NR	78%
Lampkin	1. screened at school health center	42%	NR ~68% completed at least 2 sessions	69%
	2. provider referral			
	3. clinical interview			
	4. $2500 offered to participating sites			
Librett	1. posters	~24%	67%	NA
	2. flyers			
	3. PA announcements			
	4. person-to-person			
	5. mandatory at 1 of 5 schools			
Lotecka, McWinney	1. person-to-person	78%	46% – 1 month later	NR
	2. class release time			
Matson-Koffman, Miller	1. contest with prizes	27%	NR	44%
McDonald, Roberts, Deeschaemaker	1. posters	NR	46%	NR
	2. mandatory tobacco classes			
Mills, Ewy, Dizon	1. mandatory to avoid disciplinary action	~11%	53%	53%
	2. school referral			
Murray, Prokhorov, Harty	1. state-wide campaign; 90% school participation, 95% of youth heard or saw at least 1 TV or radio ad	~90%	NA	NA
	2. funds available for programs – $0.50 per student			
Myers, Brown	In-patient facility – NA	NA	NA	78%
Myers, Brown, Kelly	1. announcements at outpatient facilities	NR	89%	80%
	2. intake interview/screening, child and parent			
Pallonen	NR – vocational high school students	NR	63% – 4 months after baseline	NA
Patten	1. sometimes flyers in schools	NR	89% – 6 months after baseline	50%
	2. sometimes press releases, TV and radio announcements			
	3. for Nicotine Dependence Center consultation			
Patterson	NR	NR	100%	100%
Pendell	NR	NR	NR	NR
Perry et al.	1. classroom program	~100%	~100%	97%
Perry et al.	1. classroom program	~100%	NR	~100%
Peters	1. widely advertised through TV and print media	94% of requesters agreed to do baseline survey; total reach NR	63%	52%
	2. free to any teen who reported smoking at least 18 months			
Peterson, Clark	1. classroom presentation	~39%	NR	100%
Popham et al.	1. state-wide campaign; 50% of youth heard or saw at least 1 TV or radio ad	NR	NA	NA
	2. youth contacted through school districts			
Prince	1. PA announcements	~6%	85%	85%
	2. person-to-person			
	3. referrals			
Quinlan and McCaul	1. screening questionnaire	66%	NA	98%
	2. ad in university newspaper			
	3. posters			
	4. extra credit or $10–15			
	5. person-to-person			
	6. lottery ($100)			
Rigotti et al.	1. written information sent from health departments	NR	NA	76% annual survey rate
	2. minor sting operation			
Skjoldbrand, Gahnberg	All youth who were seen at the clinic for check-ups, NA	100%	NA	NA
Smith et al.	1. flyers	56%	86%	77%
	2. press releases			
	3. referrals			
	4. money ($50)			
St. Pierre, Shute, Jaycox	NR	8%	100%	NA
Suedfeld et al.	1. college newspaper advertisement	NR	NR	70%
	2. screening of smokers, blind to study			
Sussman, Burton et al.	1. flyers	~9%	52%	29%
	2. PA announcements			
	3. person-to-person			
	4. class release time			
Sussman, Dent, Lichtman	1. classroom presentation	34%	54%	51%
	2. elective class credit			
	3. person-to-person			
	4. class release time			
	5. flyers			
Sussman, Dent, Stacy	1. classroom program	70%	70%	68%
	2. class credit			
Townsend et al.	1. voluntary – invitation	73%	NA	NA
Vartiainen et al.	1. campaign letter sent to schools	~3%	NA	55%
	2. youth fill out registration cards			
	3. two prizes of ~$800 at 1-month, 2 prizes of~$1,600 at 6-months			
Wakefield et al.	1. contacted school districts	80% took annual survey	NA	NA
	2. voluntary survey; strong restrictions: 57% public places, 48% home, 91% school			
Weissman et al.	1. person-to-person	NR	55%	55%
	2. voluntary – invitation			
Zavela, Harrison, Owens	1. flyers	NR	100%	100%
	2. PA announcements			
	3. ads in college newspapers			
	4. referrals			
	5. money ($20)			
Zheng	1. school staff announcements at two schools	72%	98%	NA
	2. money ($10)			

NA = not applicable; NR = not reported; ~ = approximately.

Twelve studies made use of PA announcements or classroom announcements. Eleven studies made use of screening as a means to recruit subjects. In this approach, a pool of subjects is examined through interview or file data. Those who are teen tobacco users are then asked directly to participate in a study. Ten studies made use of referrals. As defined by Sussman, Lichtman, Ritt, & Pallonen [12], referrals are similar to person-to-person recruitment in that they involve approaching particular smokers and trying to interest them or their friends in a cessation clinic. However, a person-to-person approach involved an informal means of recruitment whereas referrals involved a "push" by an official for a youth to attend a clinic. Ten studies used flyers, and seven studies used newspaper advertisements. Six studies used posters, and six studies made use of class release time. Six studies used mandatory recruitment. Students were required to attend a tobacco program either to fulfill class requirements or to avoid suspension or other negative consequences [30,54,77,82,83,89].

Five studies provided class credit, and five studies made use of TV or radio advertisements. Four studies involved presentations, in which a facilitator presented information about the project. Three studies involved assembly announcements; three studies involved policy enforcement to "place" people in a program; three studies involved administrative support; and one study each provided assistance with reminders, mentioned the program was free, or included a clinical interview (see Table 5). Multiple means of recruitment were utilized in 42 of the 61 studies that reported it.

##### Reach

Reach is defined as the number of participants who attend the first session relative to the number of adolescents notified. A total of 46 studies reported reach, or provided sufficient information such that it could be estimated, and among them a wide range exists. Reports of reach vary from six to 100%, with a mean of approximately 61%. (Reach data for studies that included mandatory attendance at programming was only available in three of six such studies and was a mean = 24%).

In 16 studies, the whole unit was treated (e.g., classrooms, school systems) such that special recruitment efforts were not necessary and participation rates were very high (mean reach = 94% of 13 studies for which reach could be calculated; see Table 5). Eleven studies made use of screening techniques to identify smokers and potential participants. Screening occurred in contexts within which the intervention was telephone based (three studies), was within a medical clinic-type setting (five studies), or was in a university setting within which administrative files or survey responses of students were screened (three studies). The mean reach was equal to 65% across eight studies for which reach could be calculated (three of these eight studies also involved whole units but are not included in the previous calculation because they made use of a screening technique).

Generally reach is calculated in school-based clinics by calculating the percentage of smokers at the school who are enrolled as participants. The mean reach could be calculated at 21 of 28 school-clinics, and it was 34%. Making brief classroom presentations by clinic facilitators seemed to show somewhat better reach (completed in five of these 21 studies, mean reach = 39%), and offering money may have improved reach (completed in two of these 21 studies, mean reach = 74%). Also, in the one study that reported it, principal encouragement led to an estimated reach of 40% of tobacco-using youths in the school [89]. Otherwise, reach did not appear to vary by type of recruitment method used in this school-based clinic context (see Table 5).

The nine studies with a reach of 15% or less were all school clinic-based, except for one very large Quit-and-Win study from Finland (3%) and a system-wide school-based Quit-and-Win study (13%). The mean number of tobacco users at baseline in the two Quit-and-Win contest studies was 3,241 and 331. The average number of tobacco users served across all studies equals 659.5 mean baseline users times a mean reach of 61%, which equals 402 subjects. Based on these overall mean data, the reach was small in these two studies, and the number of tobacco users served also was relatively small.

Of the remaining two studies in which reach was calculated, one was a hand-held computer study at a school context that relied mainly on person-to-person recruitment with some principal support (23%) [22]. The other study was a medical clinic study that involved press releases, referrals and monetary payment (53%) [80]. In summary, reach appears to be partly a function of intervention modality as opposed to type of strategy used, although there are strategies that might assist in maximizing reach within a context.

#### Retention

Posttest retention represents the percentage of tobacco users at the baseline first session that were present at the last posttest session immediately after involvement in the program and prior to follow-up data collection. Retention was not a planned statistic for 15 studies. These studies were either those that collected only pretest and follow-up data (six studies), those which made use of cross-sectional cohorts over time to assess effects of supply reduction trials, or those which involved system-wide modalities. Twelve studies failed to report retention. However, 10 of these studies did report follow-up statistics. The other two studies failed to provide any reach, retention, or follow-up statistics (see Table 5). These two studies did, however, provide quit-data [33,85]. A total of 39 studies reported retention. The range was from 33–100%, with a mean of 78%. The highest retention rates were reported in the classroom-based programs (except for the Johnson et al. [35] study, at 36%), and the lowest retention rates were reported in the school-based clinic programs.

#### Follow-up

The mean percentage of tobacco users at pretest who were present for follow-up was not reported in five studies, in which follow-up quit data were reported. Thus, for these studies, it is not clear who the subjects were that composed the quit-data. In a sixth study, follow-up data were collected but quit-rate information was not provided (odds ratios were provided [74]. In addition, follow-up data were not collected or reported in 14 studies. Of the 46 studies that did collect follow-up data, the pretest-follow-up completion rate was an average of 75% (see Table 5). Generally, highest rates of follow-up were in studies with the smallest sample sizes. For example, of the 10 studies with follow-up rates of at least 90%, sample sizes were under 100 for seven of them. The reasonably high retention and follow-up percentages indicate that cessation studies have fewer problems keeping subjects in the study than getting them enrolled. Future cessation studies possibly might best concentrate on improving reach.

Length of follow-up for the 52 studies that reported it was impressive with a mean of 8.6 months (see Table 6). Modal length of follow-up was six months (10 studies), 12 months (eight studies), three months (eight studies), five months (six studies), and one month (five studies). On average, studies exceeded the recommended adult cessation follow-up length of five months [92]. The six of eight studies with a follow-up of one month or less were pilot study clinics, in school-based or medical contexts (one was a mass media campaign and one was a smoke-free hospital study). The 15 studies with 12 months or greater follow-up included five classroom-based studies, three supply reduction studies, three community-scale studies, two medical-based clinics, one school-based clinic, and one family-based study.

**Table 6:** Cessation studies-outcomes

Investigators	% quit at posttest (Attended first session)	Mean reduction at posttest (Non-quitters/attended first session)	Length of follow-up	% quit at follow-up (Attended first session)	Mean reduction at follow-up (Non-quitters/attended first session)
Ary et al.	NR	NR	12 months	35% – program 31% – control	31%
Aveyard et al.	NR	NR	5 months (1 year after pretest)	20% – transtheoretical program 20% – control	NR
Baskerville, Hotte, Dunkley	22% – Quit-and-Win participants 6% – intervention schools 5% – control schools	NR	6 months	2% – Quit-and Win participants NR – intervention or control schools	NR
Bauman et al.	NA	NA	1 year	31% – family program 22% – control (not significant)	NR
Beaglehole et al.	NR	NR	~3 months	1% increase-program 1% decrease-control	NR
Biener et al.	NA	NA	~1 year	0%	NR – 29% decrease overall
Chakravorty	13% – both conditions	NR	NA	NA	NA
Charlton	NR	NR	6 months	17% – clinic course 10% – individual package (control)	NR
Cinnomin, Sussman	NR	NR	1 month	17% – cognitive-behavioral/social influence 0% – chemical addiction	42% – cognitive-behavioral/social influence 5% – chemical addiction
Colby et al.	NR	NR	3 months	20% – motivation interviewing 10% – brief advice	10% – both conditions
Coleman-Wallace et al.	12% – TEG 15% – TAP 0% – control (small n)	18% – TEG 24% – TAP 0% – control	NA	NA	NA
Corby et al.	100%	NA	3 weeks	0%	0%
Digiusto	8% – both program conditions	NR	3–4 months	14% – both program 7% control	NR
Dino et al.	17% – NOT, 8% brief intervention	59% – NOT, 42% – brief intervention	5 months	10% – NOT 7% – brief intervention	57% – NOT, 51% – brief intervention
Eakin, Severson, Glasgow	28%	77%	6 months	12%	45%
Etter, Ronchi, Perneger	NA	NA	7 months after baseline	0% – both program and control conditions	3% increase in program, 5% increase in control
Fibkins	19%	~30%	NA	NA	NA
Forster et al.	NA	NA	36 months	2% increase program 7% increase control	6% relative reduction
Glasgow et al.	NR	NR	6 months	10% program 6% control	NR
Glover	2%	NR	6 months	2%	NR
Goldberg, Gorn	20%	NR	3 months	11%	NR
Greenberg, Deputat	40% – fear 27% – facts 36% – values 6% – control	NR	5 months	12% – fear 16% – facts 24% – values 4% – control	NR
Hafstad, Aaro, Langmark	NR	NR	2 weeks (5 weeks since start of campaign)	12%	NR
Horn et al.	14% – NOT, 4% brief intervention	76% – NOT, 54%-brief intervention	NA	NA	NA
Horswell, Horton	~6% – Pack In Those Smokes (PITS) 0% – control	63% – PITS 0% – control	6 months	~6% – PITS 0% – control	46% – PITS 0% – control
Hotte et al.	8% – group 2% – kit-only	7% – group 3% – kit-only	6 months	4% – group NA – kit-only	4% – group NA – kit-only
Hurt et al.	11%	~49%	6 months	5%	~19%
Jason, Mollica, Ferrone	55% – both program groups 0% – control group	14% – both program groups 50% – control	17 months	41% – both program groups 0% – control	0% – both program groups 400% increase-control
Jerome	29%	41%	NA	NA	NA
Johnson et al.	0% for all groups	NR	24 months	0% for all groups	NR
Kempf, Stanley	NA	NA	2 weeks	NR – "as-if" 100% in smoke-free program but not stated 4% – program	NR
Killen et al.	NR	NR	2 months	9% – control	NR
Lampkin	NR	NR	~5 months	14%	18%
Librett	17%	39%	NA	NA	NA
Lotecka, McWhinney	NR	64% – coping 0% – information (control)	NA	NA	NA
Matson-Koffman, Miller	NR	NR	12 months	16%	~12%
McDonald, Roberts, Deeschaemaker	16%	61%	NA	NA	NA
Mills, Ewy, Dizon	15% (all were senior high youth)	8%	3 months	7%	7%
Murray, Prokhorov, Harty	2.3% – program State 0.1% increase-control State; difference not significant	NR	NA	NA	NA
Myers, Brown	NR	NR	24 months	5%	19%
Myers, Brown, Kelly	NR	NR	3 months	17%	~60%
Pallonen	~20%	NR	6 months	6%	NR
Patten et al.	18%	NR	mean of 64 months	12%	~18%
Patterson	14% – last 48 hours	NR	3 months	14%	NR
Pendell	14% across TEG and TAP	50% across TEG and TAP	NA	NA	NA
Perry et al.	NR	NR	4 months	5.7% quit (below monthly use) in program condition 4.1% increase in control condition	NR
Perry et al.	NR	NR	1–2 months	23% across conditions (27% – long-term health, 17% – social consequences, 29% – immediate effects, not significant differences, small n)	7% – long-term health, increased 16% – social consequences, 17% – immediate effects (not significant)
Peters	17% at 6 months	NR	12 months	15%	NR
Peterson, Clark	0% program and control groups	NR	1 month	0% program and control groups	44% discussion group 9% control
Popham et al.	NR	NR	12 months	2% – 1% difference as a function of exposure	NR
Prince	16%	42%	1 month	16%	42%
Quinlan, McCaul	NA	NA	1 month	3% – stage matched 14% – stage mismatched 0% – control	NR
Rigotti et al.	0% – program 1% – control	NR	24 months-anonymous surveys	3% increase-program 0% – control	NR
Skjoldebrand, Gahnberg	~4%	NR	NA	NA	NA
Smith et al.	14%	93%	12 months	5%	59%
St. Pierre, Shute, Jaycox	0%	~31%	NA	NA	NA
Suedfeld et al.	NA	NA	3 months	0%	Sensory D-Message 28% Sensory D-No Message 22% No Sensory D-Message 22% Control 0%
Sussman, Burton et al.	35% – chemical addition-ST 11% – chemical addiction – smoking 24% – psychosocial dependency – ST 26% – psychosocial dependency – smoking 8% – wait-list control-smoking	NR	3 months	15% – program conditions – ST 7% – program conditions – smoking 0% – wait list control – ST 8% – wait list control – smoking	NR
Sussman, Dent, Lichtman	14% – both program conditions NA – control	23% – both program conditions NA – control	4–5 months	17% – both program conditions 8% – control	23% – in all conditions
Sussman, Dent, Stacy	NR	NR	12 months	34% – health educator 21% – self-instruction 21% – control	15% – health educator 0% – self-instruction and control
Townsend et al.	60% – agreed to try to quit NA	NA	NA	NA	NA
Vartiainen et al.	NA	NA	6 months	28%	NR
Wakefield et al.	NA	NA	~24 months	Odds ratios Public place .90 Home ban .78 Enforce school ban .89	NA
Weisman et al.	36%	25%	5 months	36%	NR
Zavela, Harrison, Owens	NR	NR	1 month	NR	mean days abstinent mint snuff – 18.8 (63% of month) bubble gum – 21.5 (72% of month) No substitute – 23.5 (78% of month)
Zheng	13% 7-day quit rate 4% 30-day quit rate	20%	NA	NA	NA

NA = not applicable; NR = not reported; ~ = approximately.

#### Outcomes

This section reviews the outcomes of the 66 program studies. The main outcome measures are quit-rate and percentage reduction. Immediate quit-rate refers to percentage that reported quitting tobacco use, ideally for at least seven days at an immediate posttest (point prevalence abstinence), of those who attended the first session or were surveyed at baseline. An intent-to-treat approach was taken, in which those not measured at immediate posttest or at follow-up were assumed to still be using tobacco. Biochemical confirmation also was used when available. Percentage consumption reduction at immediate posttest refers to amount of reduction in tobacco use among those who did not quit at posttest. The specific statistic is the posttest level of tobacco use minus baseline level of tobacco use divided by baseline level of tobacco use. The quit-rate and percentage reduction measures are also reported at follow-up. These measures are estimated based on those who attended the first session, as well, and are calculated the same way as the immediate posttest measures.

Unfortunately, as with other data, cessation is not defined consistently. Generally, it is defined in a parallel manner to tobacco use (see Table 4). For example, if tobacco use is defined as use greater than once in the last week, cessation is defined as no use in the last week. In most studies that define tobacco use as daily use, cessation refers to no use in the last seven days or the last 30 days. (Quit results did not vary by this seven versus 30-day variation in quit-duration.) There were 16 studies in which subjects were reported to have quit tobacco but the definition of "tobacco cessation" provided is ambiguous. Four studies used the word "quit" and seemed to refer to no smoking on that day; these did use biochemical validation [18,34,41,82]. Goldberg & Gorn [54] used behavioral observation and seemed to be looking at continuous quitting. The subjects in one study were asked simply "Do you smoke cigarettes?" and two other studies mentioned that "quitting" was assessed without describing the specific assessment used [45,64,89]. Finally, eight studies reported cessation if the number of cigarettes smoked per day at posttest or follow-up was 0 [13,32,39,49,79,83,85,90].

The order of presentation of results is as follows. First, the overall level of baseline tobacco use among this sample is considered. Second, overall measures of control group cessation and reduction are estimated. Third, overall program condition cessation measures are considered. Fourth, cessation is considered as a function of program theory and modality. Fifth, cessation is considered as a function of number of program sessions. Sixth, the most effective and methodologically rigorous programs are identified. Finally, seventh, variation in effectiveness is explored as a function of gender, ethnicity, age range, and baseline tobacco use by examining the most effective program subgroup (n = 34), against the full group of programs (n = 66).

##### Baseline Tobacco Use

Level of tobacco use was defined as greater than one cigarette per week in seven studies, greater than one cigarette per month in seven studies, and simply as "current smoking" and greater than 100 lifetime cigarettes in one study. However, level of tobacco use was defined in most studies as daily use. Specifically, it was defined as at least one cigarette per day in 71% (n = 40) of the 56 studies that reported it (see Table 4). Specifically, it was defined as greater than one cigarette (or dip) per day in five studies. Also, it was defined as two to four cigarettes per day in three studies, five to nine cigarettes per day in 10 studies, 10–14 cigarettes per day in 13 studies, 15–19 cigarettes per day in seven studies, and 20 or greater cigarettes per day in three studies.

In the program studies in general, the subjects were fairly heavy smokers. An approximate grand mean of 8.4 cigarettes per day is estimated for baseline use. This mean was calculated by giving a value of once every 30 days or more a value of .033 cigarettes per day, once every seven days or more a value of .14 cigarettes per day, and current smoking a value of .14 cigarettes per day. (The estimate that current smoking is .14 may be an underestimate or an overestimate.) In addition, "greater than" statistics were estimated as being "equal to" measures. This variation in baseline tobacco use is troublesome but by making these few assumptions, one obtains a general idea of range and mean of tobacco use in cigarette per day units.

##### Consideration of Control Group Cessation and Consumption Reduction Rates

Cessation (quit) rates for the intervention studies need to be compared to cessation rates of those at comparable baseline levels of tobacco use, who have not received the programming. A strong comparison is one in which subjects can be compared directly to a randomly assigned or matched control group. That is, a control group that is measured at the same time-points as the program and that has the same baseline characteristics as the program group (i.e., in demographic composition and baseline tobacco use). The ideal comparison would be random assignment to a control group; however, when not practical, design modifications and statistical techniques can help control for possible third variable confounds [see [26]].

One other means to provide a control quit rate would be to establish an overall quit rate at a given level of tobacco use, assuming minimal program exposure. One might plot naturally occurring quit-rate as a function of baseline level of tobacco use in prospective studies. This quit-rate is likely to fluctuate over time and location, but it may be better than no use of a control quit statistic at all. Alternatively, this overall quit-rate can be determined by averaging quit-rates across all control groups in studies that do provide quasi-experimental or experimental designs. This latter type of control group quit-rate was calculated for the present review. While only a minority of the studies utilized control groups, those that did included control groups similar to the program groups, i.e., from a similar population with equivalent levels of baseline tobacco use.

Control group cessation rates at follow-up ranged from 0 to 31% in the 23 program studies that reported it (mean = 7.2%). Of these 23 studies, the breakdown of control group quit-rates is as follows. Eight studies report 0%, two studies report 1%, one study reports 4%, one study reports 6%, two studies report 7%, two studies report 8%, one study reports 9%, two studies report 10%, and one study each reports 10%, 20%, 21%, 22%, and 31%.

For three of four studies which reported a control group cessation rate of 20% or greater, baseline smoking was defined as greater than one cigarette in the last month (one study) or in the last week (two studies). Smokers were relatively young in these studies compared to other program studies. (Without those three studies composed of "light smokers" the mean cessation rate would be 5%). Thus, a control group quit rate of 7% could be considered a reasonable (even liberal) proxy for cessation in the program studies for which mean baseline smoking is at least one cigarette per day, and is a mean of approximately eight cigarettes per day, at three-to-12 months follow-up. Immediate post-program quit-rates in control groups unfortunately were reported in only 12 studies. Examining control group quit rates at follow-up, and using immediate quit-rate as a proxy if follow-up quit rate is missing (which in theory should not differ since little or no programming is offered), the mean cessation rate is 6.5% (n = 26 studies). Data is presented with and without use of this proxy measure in Tables 7 and 8.

**Table 7:** Follow-up outcomes of the 66 studies as a function of theory

Theory	# studies	Quit % (#s)	Quit 2% (#s)	% reduct (#s)	% reduct 2 (#s)
Motivation enhancement	9	19 (8)	18 (9)	27 (4)	-
Contingency-based reinforced	5	16 (5)	-	6 (2)	13 (3)
Social influences	11	12 (10)	-	27 (5)	-
Cognitive behavioral	16	11 (12)	11 (15)	32 (6)	44 (10)
Stage of change	7	11 (5)	12 (7)	13 (2)	24 (4)
Affect-oriented	2	9 (2)	-	23 (1)	-
Addiction/recovery	9	5 (3)	12 (9)	42 (4)	43 (7)
Supply reduction	7	0 (4)	0 (5)	15 (2)	-

Quit % (#s) = % quit at follow-up (# studies that provided data); Quit 2% (#s) = % quit at follow-up, and immediate posttest quit rates used if data is missing (# studies that provided data); % reduct (#s) = % reduction among non-quitters, at follow-up (# studies that provided data); % reduct 2 (#s) = % reduction among non-quitters at follow-up, and immediate posttest percentage reduction rates used if data is missing (# studies that provided data).

**Table 8:** Follow-up outcomes of the 66 studies as a function of modality

Modality	# studies	Quit % (#s)	Quit 2% (#s)	% reduct (#s)	% reduct 2 (#s)
Classroom	9	17 (9)	-	15 (4)	-
School clinics	28	12 (18)	12 (26)	34 (11)	37 (19)
Medical/recovery clinics	13	10 (9)	10 (11)	27 (8)	14 (9)
Family	1	31	-	9	-
System-wide	11	6 (9)	7 (8)	15 (2)	-
Computer	3	13 (2)	18 (3)	-	41 (1)
Sensory deprivation	1	0	-	24	-

Quit % (#s) = % quit at follow-up (# studies that provided data); Quit 2% (#s) = % quit at follow-up, and immediate posttest quit rates used if data is missing (# studies that provided data).

In several studies no one quit in the control group, whereas in other studies quit-rates hover around 7% or slightly higher. Zero quit rates in control groups generally occurred in classroom-based or system-wide modality studies (the Ary et al. study [31] is an exception, with a 31% control group quit-rate), whereas higher control group quit rates occurred in clinic studies. Those in clinic control groups generally were either on a wait-list for participation in the program, or were provided with minimal information to support cessation. One may infer that persons who are more motivated to quit – and hence seek assistance at a clinic – show higher quit-rates in a control condition. Still, as a single estimate, a 7% control group quit rate can be used as a proxy across immediate posttest and follow-up time-points.

**Percentage reduction in amount of tobacco used control group statistics **were only reported in seven studies at immediate posttest, and eight studies at follow-up. Using the follow-up measures, and using immediate posttest data as a proxy for missing data at follow-up, these data are reported in 12 studies (overall mean reduction = 10.9%). Rates reported were 0% reduction in five studies, 400% increase in one study (Jason and colleagues (1982), over a 17-month period among a very small sample of 14–16 year olds) and a 5% increase in another study (increases are assumed to be treated as 0% reduction). Also, rates were 3% reduction in one study, a 9% reduction in one study, a 23% reduction in one study, a 42% reduction in one study, and a 54% reduction in one study. As a rough, conservative rule of thumb, we can expect a percentage reduction in a control group from immediate posttest to 12 months later of 11%, particularly if offered any minimal programming. If no programming is offered, among teens it is reasonable to expect no reduction. Rather, as these teens grow older, amount of tobacco consumed is expected to increase [27].

##### Overall Program Quit Rates and Reduction

A total of 19 studies failed to report **immediate program quit rates **(i.e., right after the end of a program; generally a couple of weeks post-baseline) and 34 studies failed to report percentage reduction at immediate posttest. In addition, 10 studies did not plan to report immediate quit rates or percentage reduction by design; rather they were looking for a cumulative effect of exposure to a program or campaign over a long period of time (supply reduction or several system-wide efforts). Of the 37 studies that reported immediate program quit rates, the mean quit rate at posttest for program groups was 17.5% (range = 0 to 100%).

There were two studies with very high quit-rates and very small sample sizes. One contingency-based reinforcement study achieved 100% cessation with a sample size of eight subjects [63], which dropped off to 0% at follow-up. A second, classroom-based study (n = 32) achieved an immediate post-program quit rate of 55% [34]. Other than these two studies, the highest immediate post-program quit-rate was at 34%. A recalculation of average immediate quit rate, removing these two studies is 14.1% (n = 35 studies). Essentially, it would appear that teen cessation programs can double immediate quit rates compared to a control condition (14% versus 7%).

Of the 21 studies that did report **immediate posttest percentage consumption reduction**, mean percentage reduction was 42.6% with a very wide range across studies (range = 7% to 93%). Still, this is higher than a 0–11% control group range. Very tentatively, it would seem that programs quadruple percentage reduction compared to a control (43% versus 11%).

A total of 48 studies reported program **quit rates at follow-up**. Of the 48 studies that did provide data, an overall quit rate at time of follow-up was a mean of 11.5% (range = 0–41%; the highest quit rate was in the study by Jason and colleagues [34]. Arguably, maintenance of program effects is achieved in teen cessation studies (12% versus 7%), with a near doubling of cessation relative to controls. An examination of the six programs that required mandatory attendance revealed a mean quit-rate of 11%, which is about the same as the overall mean quit rate at follow-up.

Only 2% lower cessation overall was found at follow-up compared to immediate post-program). This result would tend to suggest that relapse rates in teen cessation studies tend to be low (15%). However, examining the 22 programs that provide quit-rate data at both immediate post-program and follow-up time-points (mean = 8.4 months follow-up), reveals a more conservative 36% relapse rate. Still, this relapse rate is much lower than with adults, which is as high as 70% over a similar time duration [27,92].

Of the 24 studies that did provide program **percentage consumption reduction data at follow-up**, the mean reduction rate was 25.8% (range = 0 to 60%). Arguably, it would seem that programs can double percentage reduction compared to a control at follow-up (26% versus 11%). For outcome data, see (see Table 6).

##### Outcomes As A Function of Theory or Modality

Differences in cessation rates as a function of theory or modality of programming is shown in Tables 7 and 8. Regarding theory-based quit-rates at follow-up, above the grand program cessation mean of 12% are the motivation enhancement (19%) and contingency-based reinforcement (16%) programs. Programs that involve manipulation of intrinsic or extrinsic motivation do the best at changing behavior over a three to 12 month follow-up period. Programs that achieved the lowest quit-rates were supply reduction (0%) and addiction/recovery-based (5%). Apparently, more impersonal programs (supply reduction), or programs that relied on use of pharmacological adjuncts or recovery from addiction themes did not work as well with teen smokers in these set of studies. Regarding percentage reduction, however, addiction/recovery-based programs, achieved relatively good results (42%; above the grand program percentage reduction mean of 26%).

Regarding program modality, classroom-based programs achieved the best quit-rates (17%). Expert system/computer-type programs (tailored self-help) also achieved high quit-rates relative to the grand program quit-rate (13%). Programming that was implemented system wide (6%), at medical-based clinics (10%) and in sensory deprivation chamber (university lab; one study, 0% quit-rate) all did relatively poorly. Programs delivered in school-based clinics (34%) and medical/recovery clinics (27%) achieved the highest percentage reductions, while other modalities did relatively poorly. Possibly, a clinic-based environment is able to encourage decreases in level of tobacco use, if not total quitting. The system-wide programs, while involving relatively more subjects, did not demonstrate a stronger overall effect (i.e., a weaker effect, but on more subjects) than did other modalities. The Wakefield et al. study [74] may provide an exception in that a 10–20% reduction in the odds of smoking was found over a very large population when access reduction was enforced; however, no quit data were provided.

One caveat with these comparisons is that they are made against a grand program mean (12%). Of course, if compared against a grand control-group mean (7%), several of these programs would seem to have tripled quit rates. A superior comparison would be to aggregate data across within-study program minus control group differences. There were far too few data points to present this analysis.

However, a few of the theory or modality sets do provide some information in this regard. For the motivation enhancement studies, the average program quit-rate minus the grand control quit rate mean of 7% is equal to 11% at follow-up. Data were available from four studies that provided program minus control group quit-rate, within-study comparisons. The average difference achieved across these studies was 8% and, if data from five studies is used (one data point from immediate posttest as a proxy), of 10%.

For the supply reduction studies, the average program quit-rate minus the grand control quit rate mean of 7% is equal to -6% at follow-up. Data were available from three studies that provided program minus control group quit-rate, within-study comparisons. A difference of 1% favoring the program condition was achieved involving three studies.

For the classroom modality programs, the average program quit-rate minus the grand control quit rate mean is equal to 10% at follow-up. Data were available from seven studies that provided program minus control group quit-rate, within-study comparisons. These studies also revealed a difference of 10%.

Likewise, a 5% difference would be expected across school clinic programs, and these exact results were achieved, involving nine studies in the program minus control group direct-comparisons. Thus, it seems that the results presented show promise in terms of accuracy.

##### Program Outcomes As A Function of Number of Sessions

Another question is whether program results vary as a function of number of sessions. Number of sessions was not indicated in two programs. For programs in which there were no sessions involved (15 programs; e.g., media or policy interventions), the follow-up quit rate was 8% and the percentage reduction was 12%. Where one to four sessions were delivered (17 programs), the follow-up quit-rate was 9% (based on 15 program data points) and the percentage reduction was 33% (based on eight program data points). Where five to eight sessions were delivered (23 programs), the follow-up quit-rate was 15% and the percentage reduction was 30% (based on 16 program data points). Where nine or more sessions were delivered (ranged from nine to 20 sessions; nine programs), the follow-up quit rate was 20% (based on eight program data points) and the percentage reduction was 46% (based on seven program data points). Clearly, number of sessions is related to program success for teen cessation.

##### Strength of Evidence of Effectiveness Provided by Studies

A table is provided that assesses the standard (quality) of evidence of effectiveness of the 34 studies that found the highest quit-rates among all 66 studies (Table 9). Evidence of effectiveness criteria are used to suggest whether or not outcomes found in a set or subset of studies provides strong, sufficient, or insufficient evidence to infer a "real" effect. This set of criteria was established by the Centers for Disease Control and is used by various research agencies to provide a first step in the process of identifying evidence-based programming (see [93]; Appendix B, Table 2).

**Table 9:** Standard of evidence of effectiveness: examining the 34 programs with the highest quit-rates

First author	Quit rate		Subjects/cell			Design				Outcome		Reach	Retain
	
	>12%	>9%	>40	>30	>20	E	QE	SG	C	IM	DIFF	24%+	49%+
Ary	*	*	*	*	*	*					4%	*	*
Aveyard	*	*	*	*	*	*					0%	*	*
Bauman	*	*	*	*	*	*					9%	*	*
Chakrovorty	*	*	*	*	*	*				*		NR	*
Charlton	*	*	*	*	*		*				7%	*	
Colby	*	*			*	*					10%	*	*
C-Wallace	*	*	*	*	*		*			*			*
Digiusto	*	*	*	*	*		*				7%		*
Dino		*	*	*	*		*				3%		*
Eakin		*			*			*				*	*
Fibkins	*	*			*			*		*			*
Glasgow		*	*	*	*	*					4%	*	*
Goldberg		*	*	*	*		*					NA	*
Greenberg	*	*			*		*				13%	*	*
Hafstad		*	*	*	*			*	*			NR	*
Horn	*	*	*	*	*		*				10%		*
Jason	*	*					*				41%	*	*
Jerome	*	*						*		*			*
Lampkin	*	*	*	*	*			*				*	*
Librett	*	*	*	*	*			*		*		*	*
M-Koffman	*	*	*	*	*			*				*	*
McDonald	*	*	*	*	*			*		*		NR	
Myers-2	*	*		*	*			*				NR	*
Patten		*	*	*	*			*				NA	*
Patterson	*	*			*			*				NR	*
Pendell	*	*	*	*	*			*		*		NR	NR
Perry-2	*	*			*	*						*	*
Peters	*	*	*	*	*			*				*	*
Prince	*	*		*	*		*						*
Sussman-2	*	*	*	*	*	*					9%	*	*
Sussman-3	*	*	*	*	*	*					13%	*	*
Vartiainen	*	*	*	*	*			*	*				*
Weissman	*	*						*				NA	*
Zheng	*	*						*		*		*	*

* = meets this criterion; NA = not applicable; NR = not reported; E = experimental design; QE = quasi-experimental design; S = single-subject design; C = community-wide type study; IM = use of an immediate outcome proxy; DIFF = whether or not and how much difference was observed between a program and a control group; + = or more.

A "strong" quality of evidence of effectiveness for a set of studies is one in which the studies were executed reasonable well. Also, the design at least includes multiple pretest or posttest measurements (if not also a comparison group), there are multiple studies used to make an inference, these studies provide consistent information, and a clinically meaningful effect size is achieved. A "sufficient" quality of evidence of effectiveness is achieved generally when the set of studies lacks one of these features. "Expert opinion" can be used as a means to judge the quality of the evidence; however, this is a relatively weak means of inference. Finally, evidence of effectiveness may be considered "insufficient." For example, if there is a great deal of difficulty with study execution (e.g., low reach, retention, follow-up), if the design suitability tends to include single pre or posttest measurements and no comparison group, if only one study is available among group of studies being examined, and if a very small effect is achieved, the evidence that a type of study is effective would be "insufficient."

Only programs that achieved a follow-up quit rate of at least 10% were included (34 of 66 studies) in the Table. Quit-rates shown in the Table are divided up between 10–12% and 13+%. The average sample sizes per cell in the design are divided into greater than 20, 30, and 40 subjects per cell. The design is experimental (E), quasi-Experimental (QE), or single group (SG). In addition, denotation of a community trial (C) was used to indicate plausible/appropriate use of a SG design (see [93]). Use of an immediate outcome proxy, which is not ideal, for a follow-up outcome is indicated by "IM." "DIFF" indicates whether or not and how much of a difference in quit-rate was observed between a program and control group. A reach of 24 + % and retention of 49 + % also are identified. If follow-up rate is 49 + %, then it too could be used as a proxy for retention rate.

Overall, the evidence of effectiveness of teen cessation programs compared to no or minimal programming is strong. The evidence of relative effectiveness for motivation enhanced and contingency-based programs are strong as well as is the evidence for use of a classroom modality. There were enough well conducted studies to make these inferences. However, the quality of the execution of these studies does vary greatly. As examples, the Ary et al. [31], Sussman, Dent, & Stacy [58], and Sussman, Dent, & Lichtman [57] programs have sample sizes greater than 40 subjects per cell, use a comparison group, do not use a proxy measure, provide a program group-control group difference score, have reach and retention rates above 23% and 48%, respectively, and achieve quit rates greater than 12%. Also, significant differences are reported between the control and program groups. The Charlton study [41] did not have a high retention, the Colby et al. [52] and Greenberg and Deputat [89] studies included a small sample size per cell. Further, the Digiusto [23] and Vartianen et al. [65] studies did not have a high reach, and the Glasgow et al. [17] study achieved only a 10% quit rate. The Murray, Prokhorov, & Harty [71] study, while achieving a 2.3% quit rate, found only a 0.1% quit rate in the control condition. (Since 450 light smokers were included in this study, apparently five people quit due to the program.)

Aside from these 34 studies, no other study achieved a quit-rate above 7%, or provided evidence against a control condition of a program effect, among the 66 programs. The criteria used are not the same as "Best Practice" criteria [see [93]]. "Best Practice" criteria provide criteria to compare teen cessation studies against each other. Within the present pool of 66 studies, no teen cessation study would show a strong or medium level of evidence of being a Best Practice (which demands multiple trials or case studies of a program). It would also be difficult to establish practicality criteria. That is, based on the research reports, it is difficult to know whether these programs vary in difficulty of facilitator training, implementation inconvenience, or cost of implementation. One can only "eyeball" these studies and get an idea on relative effectiveness of the programs that may be represented by them.

##### Outcomes As a Function of Gender and Ethnicity

Among the 34 studies with the highest program quit-rates (i.e., greater than 9%; see Table 9), 28 reported gender and, of those, 11 were composed of greater than 50% female, 13 were composed of greater than 50% male, and 4 were distributed evenly. In addition, 17 of these studies reported ethnicity and, of those, 13 were majority white and four were majority non-white. Thus, study results did not appear to vary systematically by gender and ethnicity (regarding the latter, approximately 71% of all studies were majority white). However, these data are too sparse to make any strong claims in this regard. More complete data collection and/or data reporting is needed in teen tobacco use cessation work in order to assess varying needs and responses of different groups of youth.

##### Outcomes As a Function of Age Range

Among the 34 studies with the highest program quit-rates, all reported age range. The age distribution varied from 11 to 24 years old, and modal ages were 16 years old (n = 26 studies), 17 (n = 23 studies), 15 (n = 22 studies), 18 (n = 20 studies), 14 (n = 19 studies), 13 (n = 8 studies), 19 (n = 6 studies), and 12 (n = 5 studies). Comparing the modal ages in this high-performance sub-sample (n = 34 studies) with the full sample (n = 66 studies) suggests no meaningfully significant variation in outcomes as a function of age. The sub-sample ranking matches the full sample ranking. As examples, the modal ages of 16, 17, 15, and 18 years of age are represented among 82% and 76%, 70% and 67%, 77% and 65%, and 70% and 59%, of the full sample and sub-sample, respectively.

##### Outcomes As a Function of Baseline Tobacco Use

Among the 34 studies with the highest program quit-rates, 28 reported baseline tobacco use. The mean baseline tobacco use across these studies was estimated at 8.0 cigarettes per day. Looking at only the studies that reported a quit-rate greater than 12%, the mean baseline tobacco use was estimated at 7.5 cigarettes per day (data was available on 23 studies). Since overall baseline sample tobacco use was an estimated mean of 8.4 cigarettes per day, it would appear that program success is not a function of baseline tobacco use in this set of studies.

## Self-Initiated Cessation Studies

The second part of this review pertains to all known prospective self-initiated cessation survey studies. These studies involve the collection of survey data from teen tobacco users at two or more time-points. Some baseline tobacco users report quitting at a later time-point. These ex-tobacco users are considered to exhibit "self-initiated" cessation; that is, they appeared to quit on their own without involvement in a formal quit-effort. By examining other variables measured at baseline, one can uncover predictors of later quitting (versus not quitting).

### Selection Criteria

Only prospective studies were included in this review (i.e., the same cohort of subjects are tracked over time). Ten studies were selected from a previous review-and-empirical study of self-initiated quitting [1,95-102]. Another three studies were uncovered by a search of the literature [103-105], and 4 studies provided by research colleagues [106-109].

### Study Methods

These studies involved administration of surveys at two time-points. In these studies, retention generally is over 80%, and drops to 70% over longer time lags (i.e., several years after baseline). Exceptions include that attrition (i.e., percentage of subjects who were measured at baseline but who were not measured at the second time-point) was 60% in the Stein, Newcomb, & Bentler study [98]; 33% in Sussman et al., for a high risk sample [1]; and 33% in Ary & Biglan [102]. Attrition analyses were performed in these studies indicating very few differences between those measured at two time-points and those measured only at baseline. Time lag between measurement points ranged from one month to 13 years (Table 10). Specifically, five studies provided a one year lag, two studies each provided a two year, five year, or seven year lag, and one study each provided a one month, three months, two year, four year, 12 year, or 13 year lag (the mean duration is 3.9 years). One study collected data in the 1960s, four collected data in the 1970s, 11 collected data in the 1980s, seven collected data in the 1990s, and one study collected data after the Year 2000.

**Table 10:** Summary of methods of prospective studies of self-initiated quitting among adolescents

Study authors	Duration between first and last data collection time-point	Year of first and last data time-point	% male	Age or grade range	Number of smokers	Definition of smoker	Number of quitters	% quitters	Program exposure?
Alexander et al., 1983	1 year	1979–1980	50	10–12 year old students	551	Smoked in last month; approximately 10% of the sample had smoked in the last month at pretest	242	44	Yes
Ary, Biglan, 1988	1 year	NR	50	Grades 7, 9, and 10	64	Smoked in last week	23	36	No
Chassin, Presson, Sherman, 1984	1 year	1981–1982	50	Grades 6–11	178	Monthly; 77% were weekly smokers at pretest	33	18.5	No
Chassin, Presson, Sherman, Edwards, 1991	7 years	1980–1988	50	Grades 6th through young adulthood (mean age at last wave = 21.8 years)	1056	Monthly smoking or greater; 27% of the sample were monthly smokers at pretest	338	32	No
Chen, White, Pandina, in press	7 years	1985/87–1992/94	41	18, 21, 24 year old at baseline	324	Greater than 1 cpd	57	18	No
Ellickson, McGuigan, Klein, in press	5 years	1990–1995	44	18 year old at baseline	2151	Smoked at cigarette within the last year	1093	51	Yes
Green, 1979	5 years	1974–1979	NR	12-to-18 year old at baseline	1200	NR	324	27	No
Hansen, Collins, Johnson, Graham, 1985	3 months	1981	32	15 to 16.5 yrs.	392	Identify as a "smoker" yes or no; Mean = 7 cigarettes per day at pretest	129	33	Yes
Hansen, McNeal, under review	1 year; a series of contiguous years	1993–2000	48	Grades 6–11	1894	Monthly	563	30	No
Laoye, Cresswell, Stone, 1972	2 years	1966–1968	50	Grades 7–10	375 regular smokers 830 occasional smokers	Regular – smoked every day Occasional – "once in a while"	98 regular smokers/446 of full smoker sample	26 regular smokers/37 of full smoker sample	No
Paavola, Vartiainen, Puska, in press	13 years	1978–1993	49	15–28 year old; 15 year old at baseline	183	Occasional or daily smoking; occasional smoker equals less than once per month to 1–2 times per week; approximately 29% of the sample had smoked occasionally or daily at pretest (16%, daily)	64	34 (26% of daily smokers, 46% of occasional smokers)	Yes
Sargent, Mott, Stevens, 1998	3 years	1987–1989	58	16–18 year old rural youth; 16 years old at baseline	276	Smoked in last month; approximately 20% of the sample had smoked in the last month at pretest; 55% were daily smokers	79	29	No
Skinner, Massey, Krohn, Lauer, 1985	3 years	1980–1982	50	Grades 7–12	642; perhaps 96 (15%) were weekly smokers	Smoked once or twice	96	15	No
Stein, Newcomb, Bentler, 1996	12 years	1976–1988	29	Grades 7–9 through young adulthood (mean age at last wave = 25.5 years)	18 regular 259 triers or occasional	Ever tried smoking	169	61	No
Sussman et al., unpublished data (Project TNT data)	1 year	1991–1992	50	8th grade at baseline	76	Weekly smoking; approximately 8% of the sample were weekly smokers at pretest	28	37	Yes
Sussman et al., 1998	1 year	1994–1995	58	14-to-19 year old (mean age = 16.7 years)	593	Smoked in last month; approximately 57% smoked within the last month at pretest	125	21	Yes
Zhu et al., 1999	4 years	1989–1993	53	12-to-19 year old at baseline	633	Smoked more than 100 cigarettes in life and in last 30 days; 60% were daily smokers; over all smokers mean of ~7.5 cpd	99	16	No

NA = not applicable; NR = not reported.

### Target Population

Data was collected in an overall age range extending from approximately 12 years old to 28 years old (Table 10). Baseline age ranged from 12 to 16 years old in all but one study (in Green [103], baseline age ranged from 12 to 18 years old). Gender was reported in 16 studies and was predominantly female in six studies, predominantly male in three studies, and an even split in the remaining seven studies. Ethnicity was reported in only nine studies, and the majority was white in eight of the nine studies (mean = 78.2% white; the exception was the Sussman et al. study [1]). Number of baseline smokers ranged from 64 to 2151.

### Self-initiated Study Quit Rates

Long-term self-initiated quit rates of the 17 available prospective studies averaged 31.8% (range = 15–61%; 10 of 17 studies ranged 10% around mean; 14 of 17 studies ranged 15% around mean). Three outlier study data points, 15%, 51%, and 61% – which defined smoking as "smoked once or twice," "in last year," and "ever tried" – did not measure cessation from regular use. In 6 of the studies youth had been exposed to some type of drug health programming, but not cessation material.

#### Difference in Quit-Rates: Program Studies versus Self-initiated Quit Studies

The difference in cessation rates between the program (7.2%) and self-initiated cessation (31.8%) studies may be explained in part due to baseline level of tobacco use. In the program studies, these were fairly heavy smokers. An approximate grand mean of 8.4 cigarettes per day is reported. In 16 of 17 self-initiated quitting studies that reported it, baseline smoking was defined as "ever tried" or "once or twice" in two studies. It was identified as "in the last year" in one study, at least "occasionally" in one study, "in the last 30 days" in seven studies, and "in the last seven days" in two studies. It was identified as greater than once per day in two studies; and "identify as a smoker" (a mean of seven cigarettes per day) in one study. Average smoking in the self-initiating quitting studies was approximately 0.6 cigarettes per day (having smoked once or twice is coded as .005 cigarettes per day, once in last 30 days is coded as .033 cigarettes per day, and once in last seven days is coded as .14, cigarettes per day). Clearly there was much greater variation of definition of smoking in the self-initiated quitting studies and the sample was comprised of much lighter smokers (i.e., 0.6 versus 8.4 cigarettes per day in the intervention studies). In fact, extrapolated across these mean values, for every cigarette per day increase (from 0.6 to 8.4), the percentage quit rate goes down approximately 3.2% (from 31.8 to 7.2). One could use this type of index to estimate a long-term naturally occurring quit rate among a cohort, although the variation in data across studies is great. Six self-initiated quitting studies are composed of primarily (at least 55%) daily smokers at baseline. Over a mean of 2.8 years, approximately 24% of these smokers quit. Extrapolating from this index, mean tobacco use would be approximately 2.6 cigarettes per day for these relatively heavy smokers, which does seem to fit the data (Table 11).

**Table 11:** Summary of results of prospective studies of self-initiated quitting among adolescents

Study authors	Biochemical validation?	Variables examined as predictors	Significant univariate predictors	Significant predictors controlling for covariation	Type of analysis
Alexander et al., 1983	No	Alcohol and analgesic use, friends', siblings and parental smoking, allowance, tobacco health knowledge, smoking attitudes, teacher gender, age	Greater disapproval of smoking, fewer parent, friend and sibling smokers, disapproval of cigarette advertising, lower allowance (marginal), female teacher, younger	Fewer sibling smokers, disapproval of cigarette advertising, lower allowance, younger	Logistic regression
Ary, Biglan, 1988		Pretest smoking, addiction composite, SES composite, # siblings, parent smoking and tolerance, friends' smoking, re-Yes cent and daily alcohol or marijuana use, recent cigarette offers, intention to smoke in future	Lower pretest smoking rate, less intention to smoke, fewer number of offers to smoke, fewer friends' smoking Fewer friend smokers for older youth, more negative parental attitudes for younger youth (marginal),	Higher pretest smoking	Stepwise discriminant analysis
Chassin, Presson, Sherman, 1984	Yes	Parental and friends' smoking, parental and friends' attitudes about smoking, perceived parental support, strictness of parents and friends, motivation to comply with desires of parents and friends, health beliefs, perceived control of smoking	more negative friend attitudes for younger youth, greater perceived parental support for younger youth (marginal), less strict peers (marginal), lower (higher) motivation to comply with friends for younger (older) youth (marginal)	NR	MANOVA with F and t test contrasts
Chassin, Presson, Sherman, Edwards, 1991	Yes	Parental and friends' smoking, prevalence estimates of youth and adult smoking, parental and friends' attitudes about smoking, smoking attitudes, perceived health, social, and psychological consequences of smoking, perceived parental and friends' support, health beliefs, parent and friend control over one's smoking, values agreement in one's social network, value placed on, and expectations for, academic success and independence, parents and peers expectations of one's tolerance for deviance, academic success and independence, locus of control	Less parental smoking, higher perceived parental support, higher parental expectations, greater network values agreement, more negative health and psychological concerns	NR	MANOVAs, F or t-test contrasts
Chen, White, Pandina, in press	No	Marital status, is subject a parent, work status, parental cigarette use, change in proportion of friends who smoke over the two time-points, negative beliefs about smoking, smoking to cope with stress, depression, prior heavy smoking, alcohol abuse/dependence	Married by second time point, married to a non-smoker by second time point, decreases in proportion of friends who smoke	Married by second time point, married to a nonsmoker by second time point, decreases in proportion of friends who smoke	Chi-square and logistic regression
Ellickson, McGuigan, Klein, in press	Yes	Gender, ethnicity, parental education, age, nuclear family indicator, school grades, academic intentions, cigarette refusal self-efficacy, smoking intention, friends' smoking, perceived prevalence of smoking, parents' and friends' approval of smoking, any household smoking, alcohol use, deviance, # years since first cigarette	Lower parental education, better school grades, higher refusal self-efficacy, lower smoking intention, fewer friends' use, fewer years since first cigarette	NR	Logistic regression
Green, 1979	NR	Gender, age, eight factors of smoking attitudes	Older age, less likely to hold stereotypes of smoking or of smokers	NR	NR
Hansen, Collins, Johnson, Graham, 1985	No	Friends', parents' and siblings' smoking indices; five factors established with 72 smoking attitude/belief items: negative beliefs about smoking, positive short-term consequences, social morality, normative/prevalence expectations, rebelliousness	Positive short-term consequences, social morality, friends' smoking	Desire for positive short-term consequences of smoking, society has a right to do something about smoking, fewer friends who smoke	Stepwise discriminant analysis
Hansen, McNeal, under review	No	Normative beliefs, manifest commitment, lifestyle incongruence, beliefs about consequences, resistance skill, goal setting skill, self esteem, social skill, decision making skill, stress management skill, alternatives, assistance skill	More anti-tobacco normative beliefs, manifest commitment to avoid tobacco, perceived lifestyle incongruence, more negative beliefs about consequences, resistance skill higher self esteem, slightly better decision making and stress management skills	NR	Analysis of variance
Laoye, Cresswell, Stone, 1972	No	Gender, level of smoking, grade level	More boys, occasional smokers, earlier grade levels	NR	Chi-square
Paavola, Vartiainen, Puska, in press	No	Gender, marital status, education, social class (white/blue collar), employed or not, income, children, smoking among spouse, best friend (and cessation), and co-workers, passive smoking exposure, leisure time, type/quantity of milk, fat, alcohol consumed	Female gender, married (not cohabitation), white collar, employed, spouse is nonsmoker, best friend is non-smoker, less leisure time, no milk or skim milk consumed, low or no alcohol consumption	NR	Chi-square
Sargent, Mott, Stevens, 1998	No	Gender, paternal education (SES), school performance, pretest smoking, years since initiating smoking, previous cessation attempts, attitudes toward quitting now and in future, attitudes towards heavy smokers, smoking in one's social environment, alcohol use, happiness, self-competence, locus of control, desire for independence, social awareness	Lower pretest smoking, disapproval of others = heavy smoking, male gender, less tobacco use in one's social environment in last year, no cessation experience, lack of desire to quit now, intention to quit smoking in future	Lower pretest smoking, intention to quit smoking in future	Logistic regression
Skinner, Massey, Krohn, Lauer, 1985	Yes	Attachment to father, mother, and friends, parental supervision, commitment to education, time spent on homework, commitment to work, religiosity, commitment to school activities, adherence to conventional values, morality drug use, friends' smoking, parental smoking	Fewer friends smoking for females only	Not necessary	T-tests and discriminant analysis
Stein, Newcomb, Bentler, 1996	No	Pretest smoking, depression, socializing with peers, extroversion, friends' smoking	NR	Lower pretest smoking and less friends' smoking	EQS structural equations modeling
Sussman et al., unpublished data [a]	Yes	Ethnicity, age, gender, SES, living situation, pretest smoking, smoking intention, current alcohol and marijuana use, friends' smoking and approval of smoking, prevalence estimates of peer smoking, peer commitment, refusal self-efficacy, general assertiveness, latch-key, family conflict, social maturity, risk-taking, health as a value, sense of coherence, health risk factors, self-esteem, perceived stress, loneliness/depression, program success expectancies	Greater importance of health as a value, greater sense of coherence	Greater importance of health as a value, greater sense of coherence	Logistic regression
Sussman et al., 1998	Yes	Ethnicity, age, gender, SES, living situation, acculturation, pretest smoking, smoking intention, current alcohol and marijuana and hard drug use, addiction concern, friends' smoking and approval of smoking, prevalence estimates of peer smoking, general assertiveness, family conflict, fear of victimization, morality of drug use, sensation seeking, health as a value, perceived stress, depression, program success expectancies	Latino ethnicity, not white ethnicity, lower pretest smoking, less intention to smoke in future, slightly lower addiction concern, fewer friends' who were smokers, belief in greater immorality of drug use, higher on health as a value, lower perceived stress, greater drug abuse prevention program success expectancies	Lower pretest smoking, less intention to smoke in future, lower perceived stress	Random regression model (PROC MIXED)
Zhu et al., 1999	No	Gender, age, ethnicity, smoking and quitting history, perception of danger of smoking, father, mother, sibling smoking, parental attitudes toward their children smoking, friends' smoking, school no-smoking policy, class at school on smoking health risks, school achievement, smoking intention, depression	Lower pretest smoking, never quit or quit for greater than two weeks in past, greater smoking danger perception, less intention to smoke in future, mother and father do not smoker and fewer friends' smoke, above average school achievement, less depressed	Lower pretest smoking, never quit or quit for greater than two weeks in past, less intention to smoke in future, mother not smoker, less depressed	Logistic regression

NR = not reported.

### Predictors of Quitting

Across these 17 studies, 41 significant univariate predictors were found, as is shown in Table 12. No consistent demographic trends were found. Univariate predictors in three or more studies include having fewer friends who smoke, intending not to smoke in the future, having less duration of smoking experience, having parents or siblings who do not smoke, and believing in the appropriateness of social controls against smoking. Predictors in two studies include not viewing smoking as having definite social images that are realized, holding negative outcome expectancies of smoking, and disapproving of smoking in others. Other predictors in these two studies included having greater refusal assertion skill, having higher grades, settling down (getting married, having a spouse who doesn't smoke, obtaining a job), and perceiving smoking as socially unacceptable.

**Table 12:** Significant univariate predictors considered across all studies grouped by type

Demographics	Bonding opportunities
Non-white – 1 study	Higher grades – 2 studies
Higher socioeconomic status – 1 study	Got married – 2 studies
Male gender – 2 studies, female gender – 1 study	Parental support – 2 studies
	Higher parental expectancies for child – 1 study
Behavior-related	
	Less allowance – 1 study
Low intention to smoke in future – 6 studies	Less leisure time – 1 study
Lower pretest smoking – 6 studies	Less strict peers – 1 study
Less smoking experience – 6 studies	Network values agreement – 1 study
Lower alcohol use – 1 study	Less parental education – 1 study
Better diet – 1 study	
"Not want to quit now" – 1 study	Psychology
	Less depressed – 1 study
Beliefs/attitudes toward smoking	Less perceived stress – 1 study
	Self-concern – 1 study greater, 1 lower
Higher morality/social control of tobacco use – 3 studies	
Stereotypes of smokers thwarted – 2 studies	
Negative outcome expectancies of use – 2 studies	Perceived social
Disapprove of others smoking – 2 studies	
Positive program outcome expectancies – 1 study	Fewer friends smoke – 12 studies
	No parent/sibling smoking – 4 studies
	Lower social acceptability – 2 studies
Lifestyle perceptions	
	Spouse is a non-smoker – 2 studies
High importance on health as a value – 2 studies	Parent don't like smoking – 1 study
High sense of coherence – 1 study	Fewer offers to smoke – 1 study
Perceived lifestyle incongruence – 1 study	
Life skills	
Greater refusal assertion skill – 2 studies	
Higher self-esteem – 1 study	
Better decision making skills – 1 study	
Better stress management skills – 1 study	

Directionality was aligned such that these predictors showed higher quit-rates. Each entry indicates number of studies that found this variable to be a significant predictor.

#### Controlling for Covariation Among Predictors

Seven studies did not control for co-variation among predictors. Of those 10 studies that did engage in such analyses, generally discriminant analysis or logistic regression was used. Variables found to remain significant after controlling for co-variation with other predictors included lower pretest smoking (five studies), fewer friends who smoke (four studies), lower intention to smoke in the future (three studies), and believing in the appropriateness of social controls against smoking (two studies). Other variables found to remain significant include having parents or siblings who do not smoke (two studies), having less duration of smoking experience (two studies), and not viewing smoking as having definite social images that are realized (one study). Still other variables found to remain significant include less allowance (perhaps indicating less capacity to buy cigarettes; one study), higher sense of coherence about life (one study), higher importance placed on health as a value (one study), lower perceived stress (one study), and less depression (one study). Finally, settling down was another predictor that remained significant (i.e., showing covariation with getting married between measurement waves, married to a nonsmokers; one study).

In general, the best predictors include living in a social milieu that is composed of fewer smokers (more non-smokers), intending not to smoke in the future, lower pretest smoking and less experience with smoking, belief that society should step in to place controls on smoking, perceiving smoking as negative behavior, and feeling relatively hopeful about life. Thus, social variables (this may include settling down with a nonsmoker and beginning to take on a job), intent, dependence, attitudes against smoking, and believing in a good future, perhaps, are key predictors of quitting.

## General Discussion

This paper provides the most comprehensive review to date of teen tobacco use cessation; 66 programs and 17 prospective self-initiated cessation studies were included. Detailed information from any program that provided a quit session and some data collection was included among the program studies. Also, a time lag as brief as three months was permitted for inclusion in the survey studies. Sufficient data were collected to provide at least a reasonable descriptive presentation of variables relevant to teen tobacco use cessation. For the program studies, which included heavier tobacco users than the self-initiated cessation studies, the overall control-group mean quit-rate was approximately 7% and the overall program cessation mean was 12%. Based on these data, and available studies that provided direct program-control group comparisons, there is strong evidence that teen cessation programs are more effective than doing nothing or little among those tobacco users who might attend such programs. Regarding percentage consumption reduction findings, however, there are too few studies to make any strong inferences, although it would appear that there is sufficient evidence to state that cessation programs do increase percentage reduction relative to no or little programming.

Above the grand program cessation mean of 12% are the motivation enhancement and contingency-based reinforcement theory-based programs (among eight theories). Programs that involve manipulation of intrinsic or extrinsic motivation do the best at changing behavior over a three to 12 month follow-up period.

Regarding modalities of cessation, classroom programs do the best (17% quit-rate). Three expert system (computer-type) programs showed promise (13% quit-rate). Finally, school-based clinics showed promise (12%, n = 18). In addition, it appears that program material applied more intensely (i.e., number of sessions) produces higher cessation rates. Cessation rates did not differ as a function of available data on gender, ethnicity, age, or baseline tobacco use.

It is surprising that supply reduction theory studies failed to find quit rate effects examined over multiple studies. In theory, such programs could be applied to very large numbers of youth, and even if effects were small could elicit cessation in large absolute numbers of youth. Yet, the mean quit rate was 0%. Possibly, new state-wide supply reduction efforts will indicate other findings than those shown here. One can't argue that supply reduction cessation efforts should not continue. However, exactly how these efforts can achieve cessation effects needs further investigation. Perhaps, monopolization of life contexts is needed to remove youth from opportunities for continued use [74]. On the other hand, one may argue that different types of approaches are needed to examine supply reduction effects on tobacco consumption. For example, Tauras & Chaloupka [110] used sequential longitudinal data from the Monitoring the Future Surveys, augmented with cigarette price and policy related measures to estimate smoking cessation equations among young adults (mean age = 23 years). They found that a 10% increase in prices are likely to lead to an 11–12% increase in quit rates among young male and female adults. Possibly, use of this type of methodology will reveal similar price elasticity effects on regular teen smokers, though such work is yet to be completed.

Regarding the self-initiated quit studies, behavior that seems directed away from smoking (living in a social milieu that is composed of fewer smokers, intending not to smoke in the future) is one key to cessation. Less nicotine or psychological dependence on smoking seems to be another key to youth cessation (lower pretest smoking and less experience with smoking). Anti-tobacco beliefs (e.g., that society should step in to place controls on smoking, perceiving smoking as negative behavior) also are keys to quitting. Finally, having the fortitude to maintain a quit-attempt is important (e.g., feeling relatively hopeful about life). Motivation enhancement, social skills provision, combating dependence, and achieving social support from nonsmokers are important theoretical variables that might be considered for programming. Programs that include these aspects do appear to work relatively well.

Key variables relevant to the quitting process may include gaining access and support of a context, structuring the context of programming for youth, motivating quit attempts and reducing ambivalence about quitting, making programming as enjoyable as possible, and helping youth to sustain a quit attempt (e.g., providing ongoing support during the acute withdrawal period). First, to be able to bring in the best programming possible and to facilitate access to participants, a context needs to support cessation efforts. Relevant gatekeepers can provide material support and encourage support of other staff in the same context. Certainly, inclusion of extrinsic motivators (e.g., release time) can be managed best by gatekeepers. Second, one may conjecture that programming should be tailored to the development and the lifestyles of teens. Adults are relatively likely to structure their own lives (e.g., keep careful records of their behaviors, make meeting appointments), and engage in higher-order thinking tasks (e.g., determining what "type" of smoker one is) [111]. Placed into quit-programs, the efficacy of these strategies among teens in not clear. Also, tobacco use among adults generally is at a more consistent and heavier level than teens. While use of pharmacological adjuncts are recommended for adults [3], so little work in this arena has been completed with youth that not much confidence can be placed into suggestions of the usefulness or uselessness of alternative nicotine delivery products for them. It is clear that while highly addicted youth can benefit from programming, they are less likely to quit tobacco use than are less physically addicted youth [112], and may therefore require more intensive interventions (as was recently shown in the data of Sussman, Dent, & Lichtman [57]). Some means of assisting more physically dependent youth still needs to be developed. Potentially, inpatient stays to quit tobacco might be helpful for youth, as has been completed among adults at the Mayo Clinic (the current work of R. Hurt and colleagues).

Third, programming needs to motivate quitting now rather than waiting until the future. All tobacco users should be welcome in a program, no matter what their initial stage of change is. Motivational material is likely to be helpful for most tobacco-using youth. Awareness of the changes that gradually occur as a function of smoking (e.g., increased stress, decreased mood) and quitting (e.g., decreased stress, improved mood) need instruction, along with means to help youth overcome ambivalence toward quitting [57,112].

Fourth, programming should be a fun as possible, involving games, dramatizations, and use of alternative medicine concepts. Youth will want to remain in a program that is interesting. Finally, means to support sustained quit-efforts is needed. Youth need the support of adults in multiple contexts to give them some flexibility during early quitting. Possibly, youth need to learn new social life skills so that they can reach out for the assistance they need (e.g., general conversation skills, how to use the yellow pages, knowledge of community organizations). If one was to try to coin a new theory with these steps, perhaps a "motivation, developmental tailoring, resource acquisition follow-through" model of cessation would be a possible name.

### Limitations

There are many limitations with the presentation of these data. First, several statistical means are presented without sufficient consideration of the distribution around those means. Thus, some apparent differences may not be significantly different. Provision of descriptive distributions (e.g., of control group quit-rates) does provide an indication that some differences (e.g., between program cessation and control group cessation) is clinically significant, and would be statistically significant if such methods were applied. However, it would be preferable to employ more sophisticated methods in continued work with these data. For example, use of critical values could indicate which of the theories or modalities are significantly better than the others. Also, use of multivariate methods may be able to provide some insight into the maximal combination of theory with modality for highest quit-rates.

Second, the value of the analysis is limited by the quality of the data. Numerous aggregated estimates across studies needed to be calculated to make comparisons within studies. It is much wiser to pool comparisons first made within studies. For example, an effect size, comparing the difference of a program condition to a control condition within one study should be standardized and pooled across studies to create an average effect size [e.g.[91]]. This approach is not possible with so many single-subject design studies and so many missing data points. In other words, it is difficult to conduct a meta-analysis on these cessation programs, though it could potentially provide more statistical information about precisely what works and what does not work. Only 12 and 24 studies, respectively, provided direct program-control comparisons. Thus, an absolute reduction in risk statistic also was not calculated. The use of single group designs and comparison to an overall quit rate statistic is speculative, and this important limitation needs correction through future studies that provide control groups measured concurrently with program groups.

There are many scattered areas of missing data. Ethnicity is not described in many of the studies. In studies that do describe ethnicity, a majority white sample is described. Thus, collection of ethnicity data should become a regular process in these studies, and research needs to be completed in areas with higher racial minority concentrations to assess generalizability of programming to different ethnic groups. Percentage reduction statistics are not commonly used. Thus, it is not possible to assess the totality of impact a program may have on teen tobacco use. A standard definition of baseline smoking and quitting is not used, and in several studies quitting is not measured over at least a one-week duration. Thus, it is difficult to compare studies, and the meaningfulness of cessation in several studies is suspect.

There are also several types of data that are not collected in most or all of these studies. These include the measurement of different types of tobacco use, effects of programming on other drug use, level of nicotine dependence and cessation (aside from level of pretest tobacco use), duration of smoking and cessation, and patterns of smoking and quitting among youth (i.e., across days). These other types of missing data also include provider characteristics and cessation, or matching of provider characteristics with different types of tobacco users, and issues related to cost and feasibility of implementation. Also included should be assessment of the effects of different social contexts on effectiveness of programming, measurement of mediation of program effects, and measurement of many psychosocial moderators of program effects. These additional pieces of information are needed to better understand teen tobacco use cessation.

Very recently, this review was re-examined by a team of 35 teen tobacco use cessation researchers and practitioners, sponsored by the American Legacy Foundation, Canadian Tobacco Control Research Initiative, Centers for Disease Control and Prevention, and National Cancer Institute ("Youth Tobacco Cessation Collaborative Best Practices Workshop"). The results will be presented in a guide entitled "Youth tobacco use cessation: A guide for making decision to help youth quit." These reviewers decided not to examine percentage reduction information, and made some different theoretical distinctions. In general, however, this re-analysis led to the same conclusions. There are some promising approaches as summarized herein. However, new research is needed including use of more rigorous designs.

### The Future

In 1982, Cheryl Perry wrote on the importance of developing teen cessation programming [11]. Anecdotally, many researchers and practitioners may have assumed that youth would not quit smoking until they became adults and had more reasons to quit. Many researchers were surprised to learn that youth became readily addicted to tobacco and had made several previous attempts to quit. Still, skepticism about youth cessation was based on early unsuccessful experiences.

In some of these experiences, a treatment provider acquired a quit-manual, placed a simple notice in a school or other setting, and then was surprised that only three youth showed up to the program and only one quit. There are probably numerous efforts "out there" that are not contained in this report, which relate such experiences. After more rigorous recruitment strategies were employed, more youth showed up for programming. Still, quit-rates were considered relatively low. Indeed, they are much lower than adult clinic programs, but are as high as adult minimal intervention programs [92].

Once the realization was made that youth prevention programs did not work for everyone, or that effects tended to decay, a renewed interest was gained in the promise of teen cessation programming [6,112]. The numbers of programs being researched and implemented has increased dramatically over the last 10 years. However, the technology for measuring older teen's smoking and dependence, defining cessation, and exploring various avenues of cessation assistance (e.g., use of alternative nicotine products) is brand new territory. This review is only able to summarize studies that have been completed thus far. There are many more studies currently in development or in progress (e.g., there currently are 19 teen tobacco use cessation projects underway that are being funded by the National Cancer Institute). Much more information will be learned over the next 10 years. In this time, more complete data will be collected, replication studies will be conducted, better summary analyses will be completed, and a much better understanding of teen cessation will be gained.

## Competing interests

The authors declare that they have no competing interests.
